# The impact of cumulative years of biomechanical and psychosocial exposures on competing exit routes from paid employment among older workers in Norway: A life course perspective

**DOI:** 10.5271/sjweh.4330

**Published:** 2026-11-01

**Authors:** Julie Ulstein, Karina Undem, Emil Sundstrup, Morten Birkeland Nielsen, Rachel Louise Hasting

**Affiliations:** 1 Department for Occupational Medicine and Epidemiology, National Institute of Occupational Health, Majorstuen, Oslo, Norway.; 2 National Research Centre for the Working Environment, Copenhagen, Denmark.; 3 Department for Work Psychology and Physiology, National Institute of Occupational Health, Oslo, Norway.; 4 Department of Psychosocial Science, University of Bergen, Bergen, Norway.

**Keywords:** disability pension, early retirement, health-related exit, job exposure matrix, JEM, labor market participation, unemployment

## Abstract

**Objective:**

This study aimed to examine associations between cumulative years of biomechanical and psychosocial job exposures and premature labor market exit among older workers within a competing risks framework.

**Methods:**

Using linked registry data and gender-specific job-exposure matrices, we constructed measures of cumulative years of biomechanical and psychosocial job exposures over the 30 years preceding baseline. A cohort of Norwegian workers (N=339 227) was followed from age 50 for up to 17 years, until statutory retirement age of 67 years. Fine & Gray competing risk models estimated subdistribution hazard ratios (SHR) for health-related exit, early retirement, and unemployment, stratified by gender.

**Results:**

Several exposures were positively associated with the cumulative incidence of specific exit routes. Among men, the highest SHR per additional year of exposure were observed for standing/walking for health-related exit [SHR 1.013, 95% confidence interval (CI) 1.011–1.015], low decision authority for early retirement (SHR 1.008, 95% CI 1.006–1.008), and forward bending for unemployment (SHR 1.009, 95% CI 1.005–1.013). Among women, the highest SHR were observed for standing/walking for health-related exit (SHR 1.012, 95% CI 1.010–1.015), conflicting work methods for early retirement (SHR 1.017, 95% CI 1.014–1.019), and monotonous work for unemployment (SHR 1.034, 95% CI 1.027–1.041). Several biomechanical and psychosocial exposures showed inverse associations, which could be attributable to the higher cumulative incidence of competing exit routes in the Fine & Gray framework.

**Conclusion:**

Within a competing-risks framework, cumulative years of biomechanical and psychosocial exposures were associated with distinct labor market exit patterns among older workers, highlighting the importance of incorporating work histories and competing routes into intervention design.

Despite increases in statutory retirement ages in many countries, many workers leave the labor market prematurely. In the context of declining birth rates and a declining proportion of the population within working age, early exits can contribute to labor shortages and challenge the long-term sustainability of welfare systems. Identifying the factors that contribute to premature labor market exit is therefore essential for designing policies that promote sustained participation in the workforce. Among these factors, the work environment plays a critical role, as exposure to adverse working conditions throughout working life may influence the timing and type of labor market exit later in life.

Both biomechanical and psychosocial job exposures have previously been identified as risk factors for premature labor market exit among older workers. Biomechanical exposures like years of kneeling and lifting and hard physical work have been linked to an increased risk of disability pension (1–4). Psychosocial factors like low job control and low influence at work show similar associations (1, 5, 6). Sickness absence has been linked to years of kneeling or lifting, repetitive movements, and working in awkward postures, with the risk of absence increasing progressively in line with the number of risk factors (3, 4, 7–9). For early retirement, risk factors include physically demanding work, bending or twisting of the neck and back, and working standing or squatting (4, 10–12), as well as low job control, low influence at work, and limited development opportunities (6, 11–14). Finally, for unemployment, pushing of heavy loads and bending of the back were identified as potential risk factors (15), while psychosocial risk factors include low job control and high work pace (6, 16, 17). While several psychosocial factors have been linked with an increased risk of premature labor-market exit, others appear to mitigate it (18). For example, high job demands have been identified as protective against health-related exit and early retirement (1, 5, 19).

While previous research has yielded important insights into links between biomechanical and psychosocial exposures and premature labor market exit among older workers, most studies have operationalized exposure statically (eg, at baseline), rather than considering cumulative exposure over the working life course (20). This represents a notable research gap, as cumulative exposure may increase physical and psychological strain, elevating the risk of premature labor market exit relative to exposure of shorter duration (21). Understanding the impact of cumulative job exposures is crucial for explaining older workers’ labor market participation, and for identifying which exposures to target with interventions to extend working lives. The present study aims to address this research gap by adopting a working life course perspective (22), posing the following research question: What is the association between cumulative years of biomechanical and psychosocial job exposure and premature labor market exit for older workers in the presence of competing risks?

## Methods

### Data and sample

The study sample was drawn from the Nor-Work cohort (23), linked to administrative data from Statistics Norway, including the AA-register, A-ordningen, the Norwegian population and housing censuses, the FD-trygd event database, the sickness absence register, the national education database, and the national population register. See Hasting et al (23) for a detailed description of the data sources. We define older workers as individuals aged ≥50 years, a threshold chosen because premature labor market exit in Norway increases from age 50, driven largely by disability pensions and, at older ages, early retirement (24). The sample included (i) individuals born between 1944 and 1952 registered as residents in Norway at age 50 (N=543 873), ensuring follow-up from baseline of age 50 (1994–2002) until statutory retirement age of 67 (2011–2019); (ii) individuals in paid employment at age 50 without ongoing work absence in terms of sick leave or disability pension of >50%; (iii) individuals with valid occupational codes that could be linked to job exposure matrices (JEM), without missing covariate information required for analyses. Applying these criteria led to a sample of N=339 227. A flow diagram of the selection process is presented in figure 1.

**Figure 1:**
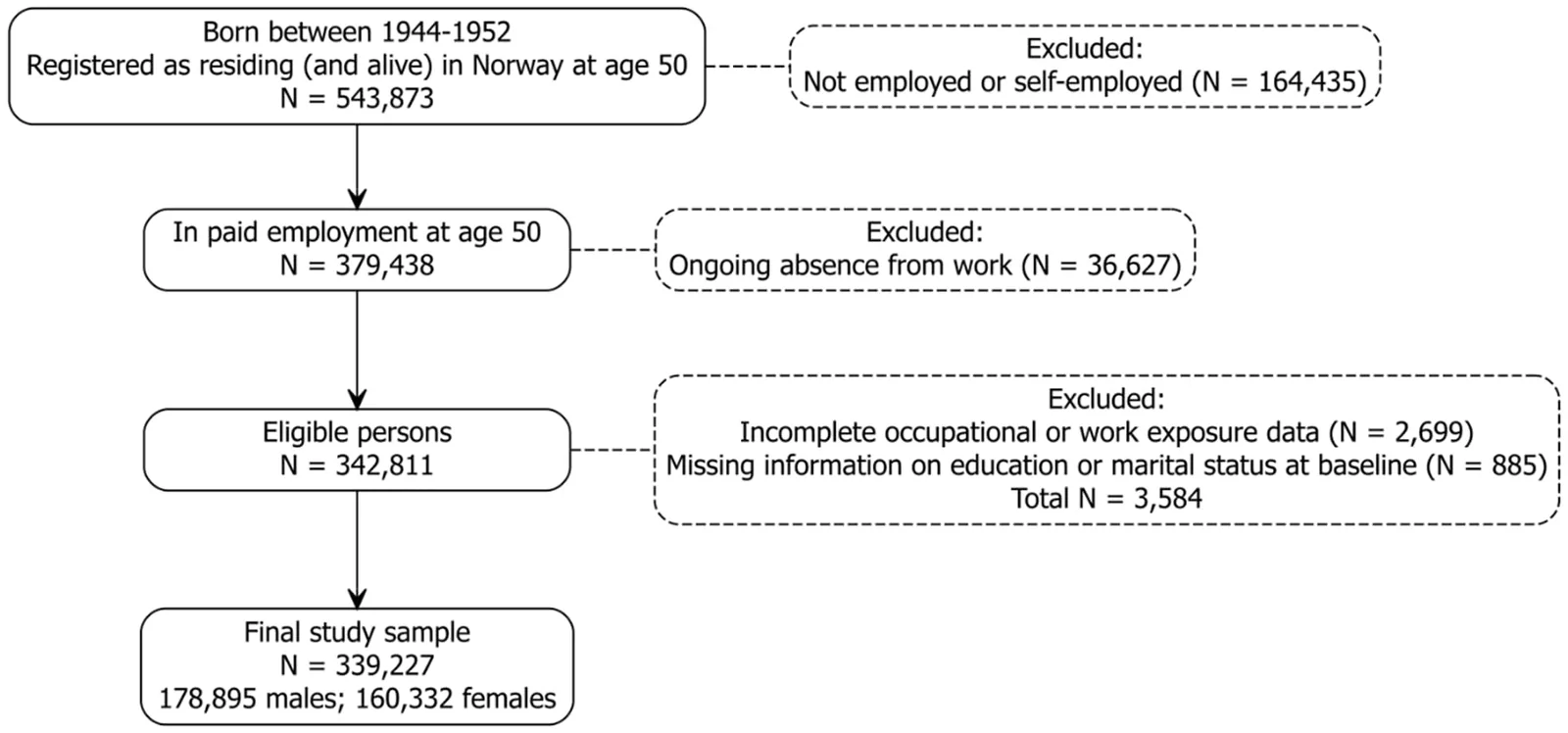
Flow diagram of study sample selection.

### Premature labor market exit

Norwegian workers can leave paid employment through several pathways. The statutory retirement age is 67, but workers can retire (partly or fully) from the age of 62, while continuing to work (partly or fully) (25). Access to early retirement depends on sufficient pension savings and the pension scheme (public or contractual pension). Another pathway is through health-related benefits, including disability pension (until statutory retirement) (26), work assessment allowance (WAA, ≤4 years) (27), and long-term sickness benefits (≤52 weeks) (28). Disability pension is granted for permanently reduced work capacity (26), whereas WAA covers temporary reduced work capacity, often following sickness absence benefits (27). A third pathway is through unemployment benefits, which can be granted for ≤2 years (29). Based on the available pathways for premature labor market exit and scientific literature (30), we defined five mutually exclusive exit routes: (i) *Health-related exit*: receiving ≥50% disability pension, alternatively receiving ≥50% sickness absence or ≥50% WAA for ≥12 months, or for <12 months if followed by ≥50% disability pension within 6 months,; with no subsequent return to work without health benefits before statutory retirement age; (ii) *Early retirement*: receiving ≥50% retirement pension and working <20 hours per week, without return to >20 hours per week before statutory retirement age; (iii) *Unemployment*: registered as fully unemployed for ≥365 days without reentry to the labor market before statutory retirement age; (iv) *Death or emigration*: registered emigration or date of death; and (v) *Economic inactivity*: no previously defined event (exit route i–iv) recorded up to the last employment record, and no subsequent qualifying event. This category may include individuals with income sources not captured in the registers (eg, private old-age and occupation-specific pensions).

If none of the defined outcomes occurred, the individuals were censored at end of follow-up. If events tied, the following hierarchy was used: death or emigration > health-related exit > early retirement > unemployed > economic inactivity.

### Biomechanical and psychosocial job exposure

Biomechanical and psychosocial job exposure was defined through two gender-specific JEM (31, 32). Both JEM are based on the Norwegian nationwide Survey of Living Conditions (NSLC) conducted in 2006, 2009, 2013, 2016, and 2019 (N=43 977), consisting of self-reported measures of biomechanical and psychosocial exposure (33). The JEM comprise 268 unique occupational groups classified according to STYRK-98, the Norwegian adaptation of the International Standard Classification of Occupations (ISCO-88). The biomechanical JEM comprises eight physical exposures: heavy lifting (>20 kg), working with hands above shoulder height, heavy physical work, neck flexion, squatting/kneeling, forward bending, awkward lifting, and standing/walking (31). The exposures represent the proportion of workers exposed in an occupation (31). We dichotomized the exposures (exposed versus unexposed) using a threshold of ≥20%, except for standing/walking ≥50% (31).

The psychosocial JEM is inspired by Karasek’s Demand–Control Model (34). The JEM incorporates four items on job demands: quantitative demands, conflicting work methods, insufficient resources, and role conflict. For job control, the JEM incorporates six items: decision authority, deciding work pace, being part of important decisions, skill utilization, skill development, and monotonous work. The exposures represent the proportion of workers exposed in an occupation (32). We dichotomized the exposures (exposed versus unexposed) using the median proportion exposed within JEM groups as the cut-off, following the scientific literature (32, 35).

### Years of job exposure

We constructed life-course exposure measures from annual work histories spanning ages 20–49 (years 1964–2001), using population censuses (1960, 1970, 1980, 1990) and employer registers from 1983 onward. The main job per year was the one with longest duration and most hours; ties were resolved by the highest occupational code. For years between censuses and before 1983, we assumed workers were employed in the same occupation for the five years ± each census (eg, the occupation in 1970 was used for the period 1966–1975). For employer register data, missing occupations were imputed by the mode occupation within industry and education strata. The yearly occupation data were merged with the JEM and the exposure variables were aggregated to years with biomechanical and psychosocial exposure (minimum=0, maximum=30 years).

### Covariates

The covariates included education (1=basic education, 2=upper secondary and tertiary vocational education, 3=education at university level); marital status at baseline (0=unmarried, divorced or widowed, 1=married or registered partner); and calendar year at baseline (1994–2002). Number of sickness absence spells ≥14 days in the two years leading up to baseline was included as a proxy to adjust for differences in baseline health.

### Statistical analysis

Fine & Gray competing risk models (36) were used to estimate associations between cumulative years of biomechanical and psychosocial exposures and each labor market exit of interest (health-related exit, early retirement, unemployment) in the presence of competing events (alternative labor market exit routes, death/emigration, and economic inactivity). We chose Fine & Gray because our objective was to estimate associations with the cumulative incidence of each labor market exit while accounting for competing events, rather than cause-specific hazards. In the Fine & Gray model, individuals who experience a competing event remain in the risk set (37), so estimates should be interpreted in light of the overall pattern across exit routes rather than in isolation. The resulting subdistribution hazard ratio (SHR) can be difficult to interpret, as it represents the instantaneous rate of the event of interest among individuals who are event-free or who have experienced a competing event and are retained in the risk set (37). Accordingly, we focus on the direction of the SHR (SHR >1 indicates higher, SHR <1 lower cumulative incidence) rather than its magnitude (37).

Prior to analyses, exposure variables were examined in terms of possible nonlinearity, pairwise correlations, and multicollinearity. Linearity on the log-SHR scale was evaluated using deviance residuals with LOESS smoothing. Residuals were obtained from gender-stratified Fine & Gray models for health-related exit, early retirement, and unemployment, adjusted for covariates. No systematic departures from linearity were observed, and the exposures were modeled as linear terms.

Pairwise correlations revealed several moderately to highly correlated exposure pairs (r>0.6; range 0.6–0.9; see supplementary material www.sjweh.fi/article/4330, table S1). To select a stable exposure set, we applied backward selection via likelihood-ratio tests: starting from the full model, we sequentially removed exposures whose exclusion did not significantly worsen fit (P>0.05), while retaining covariates. For consistency, the final exposure set was the intersection of exposures retained across models. This process removed four variables: working with hands above shoulder height, heavy physical work, deciding work pace, and being part of important decisions. We assessed multicollinearity using variance inflation factors (VIF). Among men, the highest VIF was 5.78 for neck flexion, with a mean VIF of 3.46 across all exposures. Among women, VIF were generally higher, with a maximum of 7.49 for conflicting work methods and a mean VIF of 5.87. These values indicate moderate multicollinearity but remain below the conventional threshold for severe multicollinearity (VIF ≥10). Fine & Gray models were estimated for health-related exit, early retirement, unemployment and stratified by gender. Results are reported as SHR with 95% confidence intervals (CI). Proportional SHR were assessed graphically with cumulative incidence plots, with no clear deviations observed. The analyses were conducted using the cmprsk package in R.

For sensitivity analyses, we repeated the main analyses using an alternative JEM based on the first two waves of the NSLC, in which each exposure domain has been validated (35). To assess path dependency and selective retention, we re-estimated models with the initial JEM stratified by baseline occupational class (manual versus non-manual), as limited mobility and occupation-specific skills in manual work may increase the likelihood of remaining in high-exposure roles. Finally, we modeled cumulative years of exposure categorically to assess potential non-linear impacts.

## Results

### Descriptive statistics

Table 1 reports descriptives for the full sample and by gender. Men comprised 52.7% of the sample; 52.5% had upper secondary/vocational education, and 74.2% were married. Observations were evenly distributed across the nine baseline years (10.2–12.4%). Women had a slightly higher average number of sickness absence spells [0.60, standard deviation (SD) 1.1] than men (0.40, SD 0.9). Overall, 23.9% of the sample worked until statutory retirement age (censored), followed by early retirement (23%), economic inactivity (21.8%), and health-related exit (21.7%); unemployment (4.7%) and death/emigration (4.9%) were least common. The most frequent outcomes were working until statutory retirement age for men (24.9%) and health-related exit for women (25.8%).

**Table 1:** Descriptive statistics for the study population. [SD=standard deviation. % ≥10 years=% exposed to the given factor for ≥10 years].

	Total				Men				Women		
	N (%)	Mean (SD)	% ≥10 years		N (%)	Mean (SD)	% ≥10 years		N (%)	Mean (SD)	% ≥10 years
**Categorical variables**											
Total	339 227 (100)				178 895 (52.7)				160 332 (47.3)		
Labor market exit											
Censored	80 918 (23.9)				44 583 (24.9)				36 335 (22.7)		
Health-related exit	73 677 (21.7)				32 279 (18.0)				41 398 (25.8)		
Early retirement	77 865 (23.0)				42 890 (24.0)				34 975 (21.8)		
Unemployment	16 052 (4.7)				9049 (5.1)				7003 (4.4)		
Death or emigration	16 648 (4.9)				11 064 (6.2)				5584 (3.5)		
Economic inactivity	74 067 (21.8)				39 030 (21.8)				35 037 (21.8)		
Education											
Primary education	61 088 (18.0)				30 634 (17.1)				30 454 (19.0)		
Upper secondary education	177 936 (52.5)				92 481 (51.7)				85 455 (53.3)		
Higher education	100 203 (29.5)				55 780 (31.2)				44 423 (27.7)		
Marital status											
Unmarried	87 496 (25.8)				45 151 (25.2)				42 345 (26.4)		
Married	251 731 (74.2)				133 744 (74.8)				11 7987 (73.6)		
Baseline year											
1994	37 100 (10.9)				19 856 (11.1)				17 244 (10.8)		
1995	37 774 (11.1)				19 927 (11.1)				17 847 (11.1)		
1996	42 004 (12.4)				22 281 (12.5)				19 723 (12.3)		
1997	39 891 (11.8)				20 953 (11.7)				18 938 (11.8)		
1998	38 895 (11.5)				20 578 (11.5)				18 317 (11.4)		
1999	37 223 (11.0)				19 539 (10.9)				17 684 (11.0)		
2000	36 083 (10.6)				18 913 (10.6)				17 170 (10.7)		
2001	34 762 (10.2)				18 308 (10.2)				16 454 (10.3)		
2002	35 495 (10.5)				18 540 (10.4)				16 955 (10.6)		
**Continuous variables**											
Sickness absence spells		0.5 (1.0)				0.4 (0.9)				0.6 (1.1)	
Biomechanical exposures											
Heavy lifting		11.2 (10.9)	48.6			15.3 (11.1)	64.6			6.6 (8.6)	30.7
Squatting or kneeling		9.3 (9.9)	44.4			9.6 (10.4)	44.3			9.1 (9.3)	44.3
Neck flexion		9.1 (9.8)	42.9			8.5 (10.0)	39.8			9.7 (9.6)	46.3
Forward bending		6.1 (8.6)	29.6			5.9 (8.6)	28.7			6.3 (8.5)	30.7
Awkward lifting		9.0 (9.7)	42.9			8.8 (10.0)	41.6			9.2 (9.3)	44.3
Standing or walking		13.3 (10.3)	61.3			13.2 (10.7)	59.3			13.4 (9.7)	63.5
Psychosocial exposures											
Quantitative demands		11.8 (10.5)	55.5			14.6 (10.3)	68.3			8.6 (9.7)	41.3
Conflicting work methods		11.8 (10.0)	55.8			13.4 (9.8)	64.6			10.0 (9.9)	45.9
Insufficient resources		11.8 (10.1)	55.5			13.4 (10.0)	63.5			10.0 (9.8)	46.5
Role conflict		11.7 (10.2)	55.1			14.6 (9.9)	68.4			8.5 (9.7)	40.2
Low decision authority		11.3 (9.8)	54.1			8.3 (9.0)	40.8			14.6 (9.6)	68.9
Low skill utilization		11.8 (10.0)	56.7			9.6 (9.8)	46.4			14.2 (9.7)	68.1
Low skill development		11.7 (9.7)	57.0			9.8 (9.7)	47.3			13.8 (9.2)	67.9
Monotonous work		11.5 (10.1)	54.7			10.7 (10.3)	49.9			12.4 (9.8)	60.0

Men had higher mean years of heavy lifting (15.3, SD 11.1) and psychosocial job demands (eg, quantitative demands 14.6, SD 10.3). Women had higher mean exposure to low job control (eg, low decision authority 14.6, SD 9.6). Mean years of squatting/kneeling, forward bending, awkward lifting, and standing/walking were similar between men and women, with differences <0.5 years. Large SD indicate substantial within-group variability. Overall, standing/walking was the most common ≥10-year exposure (61.3%). Among men, heavy lifting and exposures related to high job demands were most prevalent (64.6–68.4%); standing/walking and exposures related to low job control (60.0–68.9%) were most prevalent among women.

### Health-related exit

Figure 2A presents Fine & Gray estimates for the associations between cumulative years of biomechanical and psychosocial exposure and health-related exit, stratified by gender. We report estimates mutually adjusted for occupational exposures and covariates. For unadjusted estimates, see supplementary tables S2–S4. Among men, the exposures associated with a higher cumulative incidence of health-related exit were forward bending, standing/walking, low skill development, and monotonous work. The SHR ranged from 1.013 (95% CI 1.011–1.015) for standing/walking to 1.003 (95% CI 1.00–1.003) for monotonous work. Factors associated with a lower cumulative incidence included awkward lifting, quantitative demands, conflicting work methods, low decision authority, and low skill utilization, with SHR ranging from 0.993 (95% CI 0.991–0.995) for quantitative demands to 0.997 (95% CI 0.995–0.999) for low skill utilization.

Among women, the exposures associated with a higher cumulative incidence of health-related exit were forward bending, awkward lifting, standing/walking and low skill development. The SHR ranged from 1.012 (95% CI 1.010–1.015) for standing/walking to 1.003 (95% CI 1.001–1.005) for low skill development. Factors associated with a lower cumulative incidence included heavy lifting, squatting/kneeling, quantitative demands, conflicting work methods, low decision authority, low skill utilization, and monotonous work. SHR ranged from 0.992 (95% CI 0.990–0.995) for quantitative demands and low decision authority to 0.996 (95% CI 0.993–0.998) for low skill utilization.

### Early retirement

Figure 2B presents Fine & Gray estimates for the associations between cumulative years of biomechanical and psychosocial exposure and early retirement, stratified by gender. For men, exposures associated with a higher cumulative incidence of early retirement included heavy lifting, squatting/kneeling, quantitative demands, insufficient resources, low decision authority, and low skill utilization. The SHR ranged from 1.008 (95% CI 1.006–1.008) for low decision authority to 1.002 (95% CI 1.001–1.004) for squatting/kneeling. The factors associated with a lower cumulative incidence were forward bending (SHR 0.996, 95% CI 0.994–0.997) and role conflict (SHR 0.998, 95% CI 0.997–0.999).

For women, the factors associated with a higher cumulative incidence for early retirement were heavy lifting, neck flexion, conflicting work methods, insufficient resources, low decision authority, and monotonous work. The SHR ranged from 1.017 (95% CI 1.014–1.019) for conflicting work methods to 1.006 (95% CI 1.004–1.008) for low decision authority. Awkward lifting (SHR 0.990, 95% CI 0.987–0.993) was associated with a lower cumulative incidence.

### Unemployment

Figure 2C presents Fine & Gray estimates for the associations between cumulative years of biomechanical and psychosocial exposure and unemployment, stratified by gender. For men, the exposures associated with a higher cumulative incidence of unemployment included forward bending (SHR 1.009, 95% CI 1.005–1.013) and monotonous work (SHR 1.009, 95% CI 1.005–1.012). The factors associated with a lower cumulative incidence were heavy lifting, standing/walking, insufficient resources, and low skill development. The SHR ranged from 0.983 (95% CI 0.979–0.986) for insufficient resources to 0.994 (95% CI 0.991–0.997) for heavy lifting.

**Figure 2:**
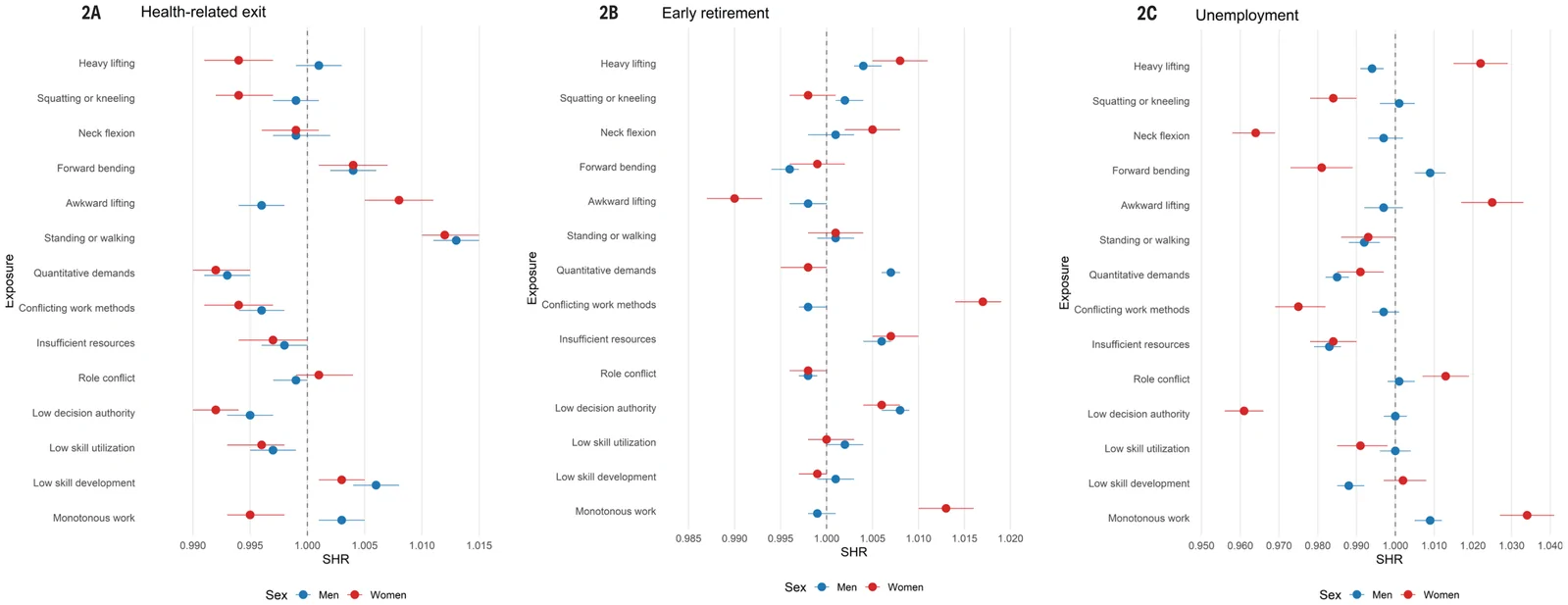
Per year associations between years of biomechanical and psychosocial exposure and (A) health-related exit, (B) early retirement. and (C) unemployment. SHR=subdistribution hazard, with 95% confidence intervals. Results mutually adjusted for biomechanical and psychosocial exposures, and for education, marital status, baseline years and number of long-term sickness absence spells leading up to baseline.

For women, the exposures associated with a higher cumulative incidence of unemployment were heavy lifting, awkward lifting, role conflict, and monotonous work. The SHR ranged from 1.034 (95% CI 1.027–1.041) for monotonous work to 1.013 (95% CI 1.007–1.019) for role conflict. The factors associated with a lower cumulative incidence included squatting/kneeling, neck flexion, forward bending, quantitative demands, conflicting work methods, insufficient resources, low decision authority, and low skill utilization. The SHR ranged from 0.961 (95% CI 0.956–0.966) for low decision authority to 0.991 (95% CI 0.985–0.997 and 0.985–0.998) for quantitative demands and low skill utilization, respectively.

### Sensitivity analyses

The supplementary analyses were largely consistent with the main results. Using the alternative JEM yielded comparable results. Stratification by occupational class did not indicate patterns of path dependency, as inverse associations appeared in both manual and non-manual occupations. Modelling cumulative years of exposure categorically produced comparable results, indicating that non-linear exposure impacts are unlikely to be driving the observed association (supplementary tables S5–S13).

## Discussion

### Summary of results

Within a competing-risks framework, this study examined how cumulative years of biomechanical and psychosocial exposures were associated with premature labor market exit, distinguishing between health-related exit, early retirement, and unemployment. Across labor market exit routes and genders, several exposures were positively associated with the cumulative incidence of specific routes, whereas others showed inverse associations.

### Interpretation of results

Biomechanical exposures were most consistently associated with higher cumulative incidence of health-related exit and early retirement among men and with health-related exit among women. Common factors for men and women were forward bending and standing/walking for health-related exit, and heavy lifting for early retirement. Some exposures were route-specific, whereas others related to multiple exits, for example, awkward lifting among women and forward bending among men were each associated with higher cumulative incidence of both health-related exit and unemployment. Several positive associations were accompanied by inverse associations for other exit routes, potentially reflecting the interdependence of outcomes within a competing-risks framework. For example, heavy lifting was associated with higher cumulative incidence of early retirement among both genders and unemployment among women, but with lower cumulative incidence of unemployment among men and health-related exit among women. The results indicate that prolonged exposure to specific biomechanical factors is associated with distinct patterns of labor market exit among men and women, suggesting that interventions tailored to specific exposures may promote sustained labor market participation among older workers. The findings are consistent with previous research linking biomechanical exposures to disability pensions and long-term sickness absence (1–4), early retirement (4, 10–12), and unemployment (15).

Cumulative years of psychosocial exposure were most consistently associated with early retirement among both men and women, with insufficient resources and low decision authority showing associations in both genders. This pattern suggests that both high job demands and low job control are associated with a higher cumulative incidence of early retirement under competing risks, and that improving psychosocial working conditions may help reduce early retirement exits among older workers. These findings are consistent with previous research on low job control (6, 11, 12, 14) but contrast research in which high job demands were associated with lower risk (19). As with the biomechanical factors, several positive associations coincided with inverse associations for alternative exit routes; for example, insufficient resources was positively associated with early retirement and inversely associated with unemployment in both genders. Monotonous work was consistently associated with health-related exit among men, early retirement among women, and unemployment among both genders, suggesting that greater task variation may help support sustained employment among older workers. Low skill development was associated with health-related exit in both genders, indicating that opportunities for learning may extend labor market participation. These findings align with prior research linking low job control to disability pension (1, 6) and unemployment (6).

Some exposures were only inversely associated with premature labor market exit, including squatting/kneeling among women and awkward lifting among men, as well as several exposures on high job demands and low control across exit routes. These associations may be partly explained by a higher cumulative incidence of the other competing exit routes, including death/emigration and economic inactivity. Alternatively, the inverse biomechanical associations may reflect that sustained, moderate exposure builds tolerance for physically demanding work, protecting against premature labor market exit, provided individual capacity is not exceeded (38). Similarly, the psychosocial inverse associations may be partly explained by the challenge-hindrance model (39), where demanding work can foster development and engagement among workers able to meet those demands. Selection mechanisms may also contribute to these patterns: Workers who remain employed at older ages despite high physical or psychological demands, or low control, may represent a particularly healthy and resilient subgroup with higher labor market attachment, reflecting a healthy worker survivor effect (40, 41). Taken together, the competing-risks framework, along with the mechanisms and selection processes noted above, may explain why some exposures were inversely associated with premature labor market exit.

The results can inform targeted interventions to mitigate the impacts of cumulative exposure on specific exit routes within a competing-risks context, thereby reducing premature labor-market exit and supporting longer working lives. More broadly, the findings may highlight the value of incorporating work history into policies and workplace practices aimed at sustaining workforce participation as workers age.

### Strengths and limitations

The strengths of the study include using high-quality registry data from Norway for the outcome variables, which allowed for long-term follow-up of older workers with no loss to follow-up and without attrition and recall bias typical for survey-based studies. The application of a competing-risks framework reflects that older workers may leave the labor market through several mutually exclusive pathways. The Fine & Gray model allowed us to estimate associations with each exit route while accounting for the occurrence of competing exits (37). Using two gender-specific JEM (31, 32), we obtained standardized biomechanical and psychosocial exposure estimates that, when linked with individual work histories, enabled the construction of accumulated years of exposure over the working career.

Exposure estimation through JEM has limitations. Assigning uniform exposure within occupational codes risks misclassification by ignoring individual and task-specific variation (42) and assumes stable exposures over the life course despite likely age-related shifts in tasks and job demands. These JEM rely on self-reported exposure data (33), which are subject to measurement error and response bias, including impacts of negative affectivity and health status (43). Consequently, JEM are likely less accurate than technical measures and combinations of self-report and expert assessments. Finally, these JEM were built from 2009–2019 data but were applied to 1964–2001 occupations, risking exposure misestimation with changing working conditions. Mechanization, automation, and assistive technologies likely reduced physical demands over time, so the JEM may underestimate biomechanical exposures for earlier periods. Conversely, post-digitalization may have increased psychological job demands through new technology, availability demands, and blurred work-life boundaries (44), potentially overestimating exposure levels when projected backwards.

Another limitation is the use of dichotomized exposure variables, which prevented differentiation of exposure levels within the exposed groups. Workers with relatively low and high levels of exposure were classified similarly, reducing exposure contrast and potentially attenuating the observed associations. This may partly explain the modest magnitude of the observed SHR.

The study design limits our ability to fully address confounding by health. We adjusted for number of sickness absence spells the two years before baseline as a proxy for baseline health. Because sickness absence may be influenced by prior exposure, this could lead to overadjustment (20). We present unadjusted results in supplementary tables S2–S4, which show that controlling for number of sickness absence spells attenuated some associations but did not change the overall pattern of results.

An additional consideration is that cumulative exposure depends on remaining employed in exposed work. Workers with longer exposure may therefore represent a selected group who were able to continue working under high-demand conditions. Consequently, the observed associations may reflect both the effects of occupational exposure and healthy worker selection (40, 41), potentially attenuating the associations toward the null or reversing their direction.

Lastly, although we mutually adjusted for biomechanical and psychosocial exposures, we did not examine how these factors jointly relate to premature labor market exit among older workers. Future studies using more detailed exposure assessment are warranted to better understand their combined contribution to premature labor market exit among older workers.

### Concluding remarks

Within a competing-risks framework, cumulative biomechanical and psychosocial work exposures were associated with distinct labor market exit routes among older workers. These findings highlight the importance of considering cumulative work histories and multiple exit routes when developing policies and workplace interventions to promote longer working lives.

## Supplementary material

Supplementary material 1 Supplementary material
