# Supplementary material for: The impact of cumulative years of biomechanical and psychosocial exposures on competing exit routes from paid employment among older workers in Norway: A life course perspective
Source: Scand J Work Environ Health. 2026 Oct 26;52(6):725–34. doi: 10.5271/sjweh.4330 (PMC13739039; doi:10.5271/sjweh.4330)
Supplement: Supplementary file 1 [file SJWEH-52-6-725-s001.pdf]

The impact of cumulative years of biomechanical and psychosocial exposures on competing exit routes from paid employment among older workers in Norway: A life course perspective[1]

by Julie Ulstein, PhD,[2] Karina Undem, PhD, Emil Sundstrup, PhD, Morten Birkeland Nielsen, PhD, Rachel Louise Hasting, PhD[1]

1. Supplementary material
2. Correspondence to: Julie Ulstein, PhD, Department for Occupational Medicine and Epidemiology, National Institute of Occupational Health, Pb 5330 Majorstuen, Oslo, 0304, Norway. [E-mail: julie.ulstein@stami.no]

Supplementary material – pairwise correlations between exposures

Table S1.Pairwise Pearson correlations between the exposure variables (continuous number of years exposed) with r >.6, separately for men and women.

**Men**			**Women**		
**Exposure**	**Exposure**	**Corr**	**Exposure**	**Exposure**	**Corr**
Awkward lifting	Standing or walking	.709	Awkward lifting	Standing or walking	.687
Conflicting work methods	Role conflict	.619	Role conflict	Decide work pace	.603
Conflicting work methods	Insufficient resources	.616	Conflicting work methods	Role conflict	.829
Decide how to go about work	Monotonous work	.657	Conflicting work methods	Decide work pace	.628
Decide work pace	Low decision authority	.615	Conflicting work methods	Insufficient resources	.847
Forward bending	Awkward lifting	.658	Low decision authority	Standing or walking	.741
Hands above shoulder height	Awkward lifting	.806	Decide work pace	Low decision authority	.762
Hands above shoulder height	Heavy physical work	.765	Decide work pace	Standing or walking	.677
Hands above shoulder height	Neck flexion	.742	Heavy lifting	Awkward lifting	.740
Hands above shoulder height	Squatting or kneeling	.916	Heavy lifting	Forward bending	.756
Hands above shoulder height	Standing or walking	.725	Heavy lifting	Heavy physical work	.699
Heavy lifting	Awkward lifting	.703	Heavy lifting	Squatting or kneeling	.613
Heavy lifting	Hands above shoulder height	.720	Heavy physical work	Awkward lifting	.616
Heavy lifting	Heavy physical work	.891	Heavy physical work	Forward bending	.823
Heavy lifting	Neck flexion	.657	Heavy physical work	Neck flexion	.657
Heavy lifting	Squatting or kneeling	.691	Heavy physical work	Squatting or kneeling	.675
Heavy lifting	Standing or walking	.661	Insufficient resources	Role conflict	.828
Heavy physical work	Awkward lifting	.679	Insufficient resources	Decide work pace	.656
Heavy physical work	Neck flexion	.651	Neck flexion	Forward bending	.724
Heavy physical work	Squatting or kneeling	.716	Neck flexion	Standing or walking	.743
Insufficient resources	Role conflict	.680	Quantitative demands	Role conflict	.647
Neck flexion	Awkward lifting	.818	Quantitative demands	Conflicting work methods	.702
Neck flexion	Forward bending	.771	Squatting or kneeling	Awkward lifting	.785
Neck flexion	Standing or walking	.700	Squatting or kneeling	Forward bending	.755
Squatting or kneeling	Awkward lifting	.837	Squatting or kneeling	Standing or walking	.675
Squatting or kneeling	Neck flexion	.755	Low skill utilization	Monotonous work	.658
Squatting or kneeling	Standing or walking	.675	Low skill utilization	Low skill development	.668
Low skill utilization	Monotonous work	.732			
Low skill utilization	Low skill development	.683			

Supplementary material – main analyses

Table S2. Associations between job exposures and health-related exit, stratified by gender

Exposure	M1, men<br>SHR (95% CI)	M2, men<br>SHR (95% CI)	M3, men<br>SHR (95% CI)	M1, women<br>SHR (95% CI)	M2, women<br>SHR (95% CI)	M3, women<br>SHR (95% CI)
Heavy lifting	1.012 (1.010–1.014)	1.002 (1.000–1.004)	1.001 (0.999–1.003)	0.995 (0.992–0.998)	0.994 (0.991–0.997)	0.994 (0.991–0.997)
Squatting or kneeling	1.004 (1.002–1.006)	0.999 (0.997–1.001)	0.999 (0.997–1.001)	0.992 (0.989–0.994)	0.994 (0.991–0.996)	0.994 (0.992–0.997)
Neck flexion	0.999 (0.997–1.002)	0.999 (0.997–1.002)	0.999 (0.997–1.002)	0.999 (0.997–1.001)	1.000 (0.998–1.002)	0.999 (0.996–1.001)
Forward bending	1.005 (1.003–1.007)	1.005 (1.003–1.007)	1.004 (1.002–1.006)	1.013 (1.009–1.016)	1.007 (1.004–1.010)	1.004 (1.001–1.007)
Awkward lifting	0.996 (0.994–0.998)	0.997 (0.995–0.999)	0.996 (0.994–0.998)	1.015 (1.012–1.018)	1.009 (1.006–1.012)	1.008 (1.005–1.011)
Standing or walking	1.007 (1.004–1.009)	1.012 (1.010–1.014)	1.013 (1.011–1.015)	1.010 (1.007–1.012)	1.013 (1.010–1.015)	1.012 (1.010–1.015)
Quantitative demands	0.989 (0.988–0.991)	0.992 (0.991–0.994)	0.993 (0.991–0.995)	0.982 (0.980–0.984)	0.991 (0.988–0.993)	0.992 (0.990–0.995)
Conflicting work methods	0.992 (0.991–0.994)	0.995 (0.993–0.996)	0.996 (0.994–0.998)	0.993 (0.990–0.996)	0.996 (0.993–0.999)	0.994 (0.991–0.997)
Insufficient resources	0.996 (0.994–0.997)	0.998 (0.996–1.000)	0.998 (0.996–1.000)	0.991 (0.989–0.994)	0.997 (0.994–0.999)	0.997 (0.994–1.000)
Role conflict	0.996 (0.994–0.998)	0.998 (0.996–1.000)	0.999 (0.997–1.000)	1.003 (1.000–1.005)	1.001 (0.999–1.003)	1.001 (0.999–1.004)
Low decision authority	0.996 (0.994–0.997)	0.994 (0.993–0.996)	0.995 (0.993–0.997)	0.985 (0.984–0.987)	0.992 (0.990–0.994)	0.992 (0.990–0.994)
Low skill utilization	0.997 (0.995–0.999)	0.996 (0.994–0.999)	0.997 (0.995–0.999)	1.001 (0.999–1.003)	0.997 (0.995–1.000)	0.996 (0.993–0.998)
Low skill development	1.008 (1.006–1.010)	1.008 (1.006–1.010)	1.006 (1.004–1.008)	1.001 (0.999–1.003)	1.003 (1.001–1.005)	1.003 (1.001–1.005)
Monotonous work	1.011 (1.009–1.013)	1.004 (1.002–1.006)	1.003 (1.001–1.005)	1.000 (0.997–1.003)	0.994 (0.991–0.997)	0.995 (0.993–0.998)
N	178895	178895	178895	160332	160332	160332

M1 – Mutually adjusted for occupational exposures. M2 – Mutually adjusted for occupational exposures, and for education, marital status and baseline year. M3 – Mutually adjusted for occupational exposures, and for education, marital status, baseline year, and long-term sickness absence spells. SHR = sub-distribution hazard, 95% CI = 95% confidence intervals. Results from M3 reported as main in the results section.

Table S3. Associations between job exposures and early retirement, stratified by gender

Exposure	M1, men SHR (95% CI)	M2, men SHR (95% CI)	M3, men SHR (95% CI)	M1, women SHR (95% CI)	M2, women SHR (95% CI)	M3, women SHR (95% CI)
Heavy lifting	1.004 (1.002–1.005)	1.004 (1.003–1.006)	1.004 (1.003–1.006)	1.007 (1.004–1.010)	1.007 (1.005–1.010)	1.008 (1.005–1.011)
Squatting or kneeling	1.002 (1.000–1.004)	1.002 (1.000–1.004)	1.002 (1.001–1.004)	0.998 (0.996–1.001)	0.998 (0.996–1.001)	0.998 (0.996–1.001)
Neck flexion	1.002 (0.999–1.004)	1.001 (0.998–1.003)	1.001 (0.998–1.003)	1.004 (1.002–1.007)	1.004 (1.002–1.007)	1.005 (1.002–1.008)
Forward bending	0.995 (0.993–0.997)	0.995 (0.993–0.997)	0.996 (0.994–0.997)	0.997 (0.994–1.000)	0.998 (0.995–1.001)	0.999 (0.996–1.002)
Awkward lifting	0.998 (0.996–1.000)	0.998 (0.996–1.000)	0.998 (0.996–1.000)	0.989 (0.986–0.992)	0.990 (0.987–0.993)	0.990 (0.987–0.993)
Standing or walking	1.001 (1.000–1.003)	1.001 (0.999–1.003)	1.001 (0.999–1.003)	1.002 (0.999–1.005)	1.001 (0.998–1.004)	1.001 (0.998–1.004)
Quantitative demands	1.008 (1.007–1.010)	1.007 (1.006–1.009)	1.007 (1.006–1.008)	0.999 (0.997–1.002)	0.998 (0.996–1.001)	0.998 (0.995–1.000)
Conflicting work methods	0.999 (0.997–1.001)	0.999 (0.997–1.000)	0.998 (0.997–1.000)	1.017 (1.014–1.020)	1.016 (1.013–1.019)	1.017 (1.014–1.019)
Insufficient resources	1.006 (1.005–1.008)	1.006 (1.004–1.007)	1.006 (1.004–1.007)	1.009 (1.006–1.011)	1.008 (1.005–1.011)	1.007 (1.005–1.010)
Role conflict	0.997 (0.996–0.999)	0.998 (0.997–1.000)	0.998 (0.997–0.999)	0.997 (0.995–0.999)	0.998 (0.996–1.001)	0.998 (0.996–1.000)
Low decision authority	1.008 (1.007–1.010)	1.008 (1.006–1.009)	1.008 (1.006–1.009)	1.009 (1.007–1.011)	1.007 (1.005–1.009)	1.006 (1.004–1.008)
Low skill utilization	1.003 (1.001–1.005)	1.002 (1.001–1.004)	1.002 (1.000–1.004)	0.999 (0.996–1.001)	1.000 (0.997–1.002)	1.000 (0.998–1.003)
Low skill development	0.999 (0.997–1.001)	1.000 (0.998–1.002)	1.001 (0.999–1.003)	0.999 (0.997–1.001)	0.999 (0.997–1.001)	0.999 (0.997–1.000)
Monotonous work	0.999 (0.997–1.001)	0.999 (0.998–1.001)	0.999 (0.998–1.001)	1.011 (1.009–1.014)	1.013 (1.011–1.016)	1.013 (1.010–1.016)
N	178895	178895	178895	160332	160332	160332

M1 – Mutually adjusted for occupational exposures. M2 – Mutually adjusted for occupational exposures, and for education, marital status and baseline year. M3 – Mutually adjusted for occupational exposures, and for education, marital status, baseline year, and long-term sickness absence spells. SHR = sub-distribution hazard, 95% CI = 95% confidence intervals. Results from M3 reported as main in the results section.

Table S4. Associations between job exposures and unemployment, stratified by gender

Exposure	M1, men<br>SHR (95% CI)	M2, men<br>SHR (95% CI)	M3, men<br>SHR (95% CI)	M1, women<br>SHR (95% CI)	M2, women<br>SHR (95% CI)	M3, women<br>SHR (95% CI)
Heavy lifting	1.005 (1.002–1.008)	0.994 (0.991–0.997)	0.994 (0.991–0.997)	1.027 (1.020–1.034)	1.022 (1.015–1.029)	1.022 (1.015–1.029)
Squatting or kneeling	1.004 (1.000–1.008)	1.001 (0.996–1.005)	1.001 (0.996–1.005)	0.984 (0.977–0.990)	0.984 (0.978–0.990)	0.984 (0.978–0.990)
Neck flexion	0.997 (0.992–1.002)	0.997 (0.993–1.002)	0.997 (0.993–1.002)	0.960 (0.954–0.966)	0.963 (0.958–0.969)	0.964 (0.958–0.969)
Forward bending	1.009 (1.005–1.013)	1.009 (1.005–1.013)	1.009 (1.005–1.013)	0.984 (0.976–0.992)	0.980 (0.972–0.989)	0.981 (0.973–0.989)
Awkward lifting	0.994 (0.990–0.999)	0.997 (0.992–1.002)	0.997 (0.992–1.002)	1.031 (1.023–1.040)	1.025 (1.017–1.033)	1.025 (1.017–1.033)
Standing or walking	0.987 (0.983–0.991)	0.992 (0.988–0.996)	0.992 (0.988–0.996)	0.991 (0.984–0.997)	0.993 (0.986–1.000)	0.993 (0.986–1.000)
Quantitative demands	0.980 (0.977–0.982)	0.985 (0.982–0.988)	0.985 (0.982–0.988)	0.985 (0.980–0.991)	0.991 (0.986–0.997)	0.991 (0.985–0.997)
Conflicting work methods	0.995 (0.992–0.998)	0.997 (0.994–1.001)	0.997 (0.994–1.001)	0.977 (0.970–0.983)	0.975 (0.968–0.982)	0.975 (0.969–0.982)
Insufficient resources	0.981 (0.978–0.984)	0.983 (0.979–0.986)	0.983 (0.979–0.986)	0.976 (0.970–0.982)	0.984 (0.978–0.990)	0.984 (0.978–0.990)
Role conflict	0.999 (0.995–1.002)	1.001 (0.998–1.005)	1.001 (0.998–1.005)	1.012 (1.006–1.018)	1.013 (1.007–1.019)	1.013 (1.007–1.019)
Low decision authority	1.005 (1.003–1.008)	1.000 (0.997–1.003)	1.000 (0.997–1.003)	0.956 (0.951–0.961)	0.961 (0.956–0.966)	0.961 (0.956–0.966)
Low skill utilization	1.002 (0.998–1.006)	1.000 (0.996–1.004)	1.000 (0.996–1.004)	0.996 (0.989–1.002)	0.991 (0.984–0.998)	0.991 (0.985–0.998)
Low skill development	0.987 (0.983–0.991)	0.988 (0.985–0.992)	0.988 (0.985–0.992)	1.000 (0.995–1.005)	1.002 (0.997–1.008)	1.002 (0.997–1.008)
Monotonous work	1.011 (1.007–1.014)	1.009 (1.006–1.012)	1.009 (1.005–1.012)	1.039 (1.032–1.046)	1.034 (1.027–1.041)	1.034 (1.027–1.041)
N	178895	178895	178895	160332	160332	160332

M1 – Mutually adjusted for occupational exposures. M2 – Mutually adjusted for occupational exposures, and for education, marital status and baseline year. M3 – Mutually adjusted for occupational exposures, and for education, marital status, baseline year, and long-term sickness absence spells. SHR = sub-distribution hazard, 95% CI = 95% confidence intervals. Results from M3 reported as main in the results section.

Supplementary material – sensitivity analyses

Sensitivity analyses using alternative Norwegian JEM

The tables below present the results from similar analyses using an alternative JEM, where each exposure domain has been validated (Hanvold et al., 2019). The group-based exposure estimates, defined as the proportion of workers within each group who were exposed, were dichotomized using the same cut-off levels as in the validation article. For biomechanical exposures, heavy physical work, working with arms raised, and heavy lifting were dichotomized at 20%, awkward lifting, squatting/kneeling, and working with head bending were dichotomized at 30%, while standing/walking was dichotomized at 50%. The psychosocial exposures were dichotomized at the median, where high psychological demands consisted of quantitative demands, role conflict and emotional demands, and low decision latitude consisted of decision authority and skill discretion.

Table S5. Associations between job exposures and health-related exit, stratified by gender

**Exposure**	**M1, men**	**M2, men**	**M3, men**	**M1, women**	**M2, women**	**M3, women**
	SHR (95% CI)	SHR (95% CI)	SHR (95% CI)	SHR (95% CI)	SHR (95% CI)	SHR (95% CI)
Heavy physical work	1.009 (1.007–1.011)	1.005 (1.003–1.007)	1.003 (1.001–1.005)	1.014 (1.012–1.017)	1.006 (1.004–1.008)	1.003 (1.001–1.006)
Squatting/kneeling	1.002 (1.000–1.004)	1.002 (1.000–1.005)	1.003 (1.001–1.005)	0.993 (0.991–0.995)	0.995 (0.993–0.997)	0.994 (0.991–0.996)
Arms raised	0.997 (0.995–0.999)	0.996 (0.994–0.998)	0.997 (0.995–0.999)	1.010 (1.008–1.012)	1.005 (1.003–1.007)	1.007 (1.004–1.009)
Standing/walking	1.009 (1.007–1.011)	1.012 (1.010–1.014)	1.011 (1.009–1.013)	1.013 (1.011–1.015)	1.015 (1.013–1.017)	1.013 (1.011–1.015)
Awkward lifting	0.999 (0.997–1.001)	0.998 (0.996–1.000)	0.998 (0.996–1.001)	0.998 (0.995–1.001)	1.001 (0.999–1.004)	1.000 (0.997–1.003)
Head bending	0.993 (0.991–0.996)	0.996 (0.994–0.998)	0.996 (0.993–0.998)	0.997 (0.995–0.999)	0.999 (0.997–1.001)	0.999 (0.997–1.001)
Heavy lifting	1.010 (1.008–1.012)	1.002 (0.999–1.004)	1.001 (0.999–1.003)	1.000 (0.997–1.002)	0.996 (0.994–0.999)	0.996 (0.994–0.999)
Forward bending	1.007 (1.004–1.010)	1.007 (1.005–1.010)	1.006 (1.004–1.009)	1.018 (1.016–1.021)	1.008 (1.006–1.011)	1.008 (1.005–1.010)
High psychological demands	0.991 (0.989–0.992)	0.996 (0.995–0.998)	0.997 (0.995–0.999)	0.975 (0.973–0.977)	0.988 (0.986–0.989)	0.988 (0.986–0.989)
Low decision latitude	1.004 (1.002–1.005)	1.004 (1.003–1.006)	1.004 (1.003–1.006)	0.995 (0.993–0.997)	0.997 (0.995–0.999)	0.997 (0.995–0.999)
Monotonous work	1.009 (1.007–1.010)	1.001 (1.000–1.003)	1.001 (1.000–1.003)	0.988 (0.986–0.990)	0.987 (0.985–0.989)	0.988 (0.986–0.989)
N	178895	178895	178895	160332	160332	160332

M1 – Mutually adjusted for occupational exposures. M2 – Mutually adjusted for occupational exposures, and for education, marital status and baseline year. M3 – Mutually adjusted for occupational exposures, and for education, marital status, baseline year, and long-term sickness absence spells. SHR = sub-distribution hazard, 95% CI = 95% confidence intervals.

Table S6. Associations between job exposures and early retirement, stratified by gender

Exposure	M1, men<br>SHR (95% CI)	M2, men<br>SHR (95% CI)	M3, men<br>SHR (95% CI)	M1, women<br>SHR (95% CI)	M2, women<br>SHR (95% CI)	M3, women<br>SHR (95% CI)
Heavy physical work	0.997 (0.995–0.999)	0.998 (0.997–1.000)	0.999 (0.997–1.001)	0.997 (0.994–1.000)	1.000 (0.997–1.002)	1.001 (0.998–1.004)
Squatting/kneeling	1.007 (1.005–1.009)	1.005 (1.003–1.007)	1.005 (1.003–1.007)	1.015 (1.013–1.018)	1.014 (1.011–1.016)	1.014 (1.011–1.017)
Arms raised	1.001 (0.999–1.003)	1.001 (0.999–1.003)	1.000 (0.999–1.002)	0.984 (0.982–0.987)	0.986 (0.984–0.989)	0.986 (0.983–0.988)
Standing/walking	0.998 (0.996–0.999)	0.999 (0.997–1.000)	0.999 (0.998–1.001)	0.999 (0.997–1.002)	0.999 (0.997–1.001)	1.000 (0.998–1.002)
Awkward lifting	0.994 (0.992–0.996)	0.995 (0.993–0.997)	0.995 (0.993–0.997)	1.004 (1.001–1.006)	1.003 (1.000–1.005)	1.003 (1.000–1.006)
Head bending	1.001 (0.999–1.003)	1.000 (0.998–1.002)	1.000 (0.998–1.002)	0.995 (0.993–0.997)	0.995 (0.993–0.997)	0.995 (0.993–0.997)
Heavy lifting	1.004 (1.002–1.006)	1.003 (1.001–1.005)	1.003 (1.001–1.005)	1.004 (1.001–1.006)	1.005 (1.002–1.007)	1.005 (1.002–1.007)
Forward bending	0.996 (0.993–0.998)	0.996 (0.994–0.998)	0.996 (0.994–0.999)	0.981 (0.978–0.984)	0.983 (0.981–0.986)	0.984 (0.981–0.986)
High psychological demands	1.003 (1.002–1.004)	1.003 (1.001–1.004)	1.003 (1.001–1.004)	1.011 (1.009–1.013)	1.009 (1.007–1.011)	1.009 (1.007–1.010)
Low decision latitude	1.007 (1.006–1.008)	1.007 (1.006–1.008)	1.007 (1.006–1.008)	1.016 (1.014–1.018)	1.015 (1.014–1.017)	1.015 (1.013–1.017)
Monotonous work	1.000 (0.999–1.002)	1.000 (0.999–1.001)	1.000 (0.999–1.001)	1.007 (1.005–1.009)	1.008 (1.006–1.010)	1.008 (1.006–1.009)
N	178895	178895	178895	160332	160332	160332

M1 – Mutually adjusted for occupational exposures. M2 – Mutually adjusted for occupational exposures, and for education, marital status and baseline year. M3 – Mutually adjusted for occupational exposures, and for education, marital status, baseline year, and long-term sickness absence spells. SHR = sub-distribution hazard, 95% CI = 95% confidence intervals.

Table S7. Associations between job exposures and unemployment, stratified by gender

Exposure	M1, men<br>SHR (95% CI)	M2, men<br>SHR (95% CI)	M3, men<br>SHR (95% CI)	M1, women<br>SHR (95% CI)	M2, women<br>SHR (95% CI)	M3, women<br>SHR (95% CI)
Heavy physical work	1.005 (1.001–1.009)	1.001 (0.997–1.005)	1.001 (0.997–1.005)	1.028 (1.022–1.033)	1.021 (1.015–1.026)	1.021 (1.016–1.027)
Squatting/kneeling	0.993 (0.988–0.997)	0.993 (0.988–0.997)	0.993 (0.988–0.997)	0.949 (0.943–0.955)	0.948 (0.942–0.954)	0.948 (0.942–0.954)
Arms raised	0.992 (0.988–0.997)	0.991 (0.987–0.995)	0.991 (0.987–0.995)	1.032 (1.028–1.037)	1.032 (1.027–1.037)	1.032 (1.027–1.037)
Standing/walking	0.993 (0.989–0.996)	0.993 (0.989–0.996)	0.993 (0.989–0.996)	0.987 (0.982–0.991)	0.984 (0.979–0.989)	0.984 (0.979–0.989)
Awkward lifting	1.015 (1.011–1.020)	1.015 (1.010–1.020)	1.015 (1.010–1.020)	1.001 (0.994–1.007)	1.003 (0.997–1.010)	1.003 (0.997–1.010)
Head bending	0.997 (0.992–1.002)	0.999 (0.995–1.004)	0.999 (0.995–1.004)	0.974 (0.969–0.979)	0.979 (0.974–0.984)	0.979 (0.974–0.984)
Heavy lifting	1.006 (1.003–1.010)	1.000 (0.996–1.004)	1.000 (0.996–1.004)	1.016 (1.011–1.021)	1.015 (1.009–1.020)	1.015 (1.009–1.020)
Forward bending	1.003 (0.998–1.009)	1.005 (0.999–1.010)	1.005 (0.999–1.010)	1.021 (1.015–1.027)	1.012 (1.006–1.018)	1.012 (1.006–1.018)
High psychological demands	0.978 (0.975–0.982)	0.987 (0.984–0.990)	0.987 (0.984–0.990)	0.955 (0.951–0.959)	0.967 (0.963–0.972)	0.967 (0.963–0.972)
Low decision latitude	0.988 (0.986–0.991)	0.989 (0.986–0.992)	0.989 (0.986–0.992)	0.967 (0.964–0.971)	0.970 (0.966–0.973)	0.970 (0.966–0.974)
Monotonous work	1.012 (1.009–1.014)	1.004 (1.001–1.006)	1.004 (1.001–1.006)	1.016 (1.012–1.020)	1.011 (1.007–1.016)	1.011 (1.006–1.016)
N	178895	178895	178895	160332	160332	160332

M1 – Mutually adjusted for occupational exposures. M2 – Mutually adjusted for occupational exposures, and for education, marital status and baseline year. M3 – Mutually adjusted for occupational exposures, and for education, marital status, baseline year, and long-term sickness absence spells. SHR = sub-distribution hazard, 95% CI = 95% confidence intervals.

Sensitivity analyses with stratification by occupational class

Table S8. Associations between job exposures and health-related exit, stratified by gender and occupational class at baseline

	**Men**				**Women**			
	**M1**	**M2**	**M1**	**M2**	**M1**	**M2**	**M1**	**M2**
	**Non-manual**	**Non-manual**	**Manual**	**Manual**	**Non-manual**	**Non-manual**	**Manual**	**Manual**
**Exposure**	SHR (95% CI)	SHR (95% CI)	SHR (95% CI)	SHR (95% CI)	SHR (95% CI)	SHR (95% CI)	SHR (95% CI)	SHR (95% CI)
Heavy lifting	1.003 (1.000–1.006)	1.001 (0.998–1.004)	0.991 (0.989–0.993)	0.994 (0.992–0.996)	0.986 (0.982–0.991)	0.989 (0.985–0.993)	0.989 (0.985–0.992)	0.994 (0.990–0.998)
Squatting or kneeling	1.003 (0.999–1.007)	1.001 (0.998–1.005)	0.991 (0.989–0.993)	0.994 (0.992–0.997)	1.001 (0.996–1.005)	0.999 (0.994–1.004)	0.986 (0.982–0.991)	0.994 (0.989–0.999)
Neck flexion	0.996 (0.992–0.999)	0.998 (0.995–1.002)	0.993 (0.991–0.995)	0.995 (0.992–0.997)	0.994 (0.989–0.998)	0.998 (0.994–1.003)	1.002 (0.998–1.006)	0.998 (0.994–1.003)
Forward bending	0.983 (0.980–0.986)	0.991 (0.988–0.994)	1.001 (0.999–1.004)	1.002 (0.999–1.004)	1.001 (0.997–1.005)	1.003 (0.999–1.007)	1.002 (0.998–1.006)	0.999 (0.995–1.002)
Awkward lifting	1.000 (0.997–1.003)	0.998 (0.994–1.001)	1.001 (0.998–1.003)	1.000 (0.998–1.003)	0.991 (0.988–0.994)	0.994 (0.991–0.997)	0.984 (0.980–0.987)	0.989 (0.985–0.992)
Standing or walking	1.008 (1.003–1.012)	1.005 (1.001–1.009)	0.994 (0.991–0.997)	0.997 (0.994–1.000)	1.000 (0.996–1.004)	0.997 (0.993–1.001)	0.996 (0.992–1.001)	0.996 (0.992–1.000)
Quantitative demands	1.010 (1.006–1.015)	1.009 (1.005–1.014)	1.001 (0.999–1.004)	0.998 (0.996–1.001)	1.002 (0.999–1.005)	1.002 (0.999–1.005)	1.009 (1.005–1.014)	1.008 (1.003–1.012)
Conflicting work methods	1.000 (0.996–1.004)	0.994 (0.991–0.998)	1.010 (1.008–1.013)	1.005 (1.002–1.007)	1.006 (1.001–1.010)	0.999 (0.994–1.004)	0.995 (0.991–0.998)	0.994 (0.990–0.998)
Insufficient resources	1.003 (0.999–1.006)	0.998 (0.994–1.002)	1.011 (1.008–1.014)	1.006 (1.003–1.009)	0.998 (0.993–1.003)	0.995 (0.990–1.001)	0.992 (0.988–0.996)	0.994 (0.990–0.998)
Role conflict	1.006 (1.001–1.011)	1.000 (0.995–1.005)	1.002 (1.000–1.005)	0.999 (0.997–1.002)	0.990 (0.986–0.995)	0.991 (0.986–0.995)	0.988 (0.984–0.992)	0.991 (0.987–0.995)
Low decision authority	0.997 (0.992–1.003)	0.996 (0.991–1.002)	1.000 (0.997–1.003)	1.002 (0.999–1.005)	1.010 (1.005–1.014)	1.008 (1.003–1.012)	0.990 (0.987–0.994)	0.991 (0.987–0.995)
Low skill utilization	1.001 (0.996–1.006)	1.001 (0.996–1.007)	1.005 (1.003–1.007)	1.004 (1.002–1.006)	1.001 (0.995–1.006)	1.000 (0.994–1.006)	1.017 (1.012–1.021)	1.010 (1.005–1.014)
Low skill development	1.000 (0.994–1.006)	0.999 (0.994–1.005)	0.993 (0.991–0.996)	0.994 (0.991–0.996)	1.017 (1.011–1.022)	1.014 (1.008–1.019)	1.013 (1.008–1.017)	1.008 (1.004–1.013)
Monotonous work	1.015 (1.011–1.019)	1.017 (1.013–1.022)	1.000 (0.996–1.003)	1.003 (1.000–1.006)	1.002 (0.997–1.006)	1.006 (1.001–1.010)	1.013 (1.008–1.018)	1.013 (1.008–1.018)
N	178895	178895	178895				160332	160332

M1 – Mutually adjusted for occupational exposures. M2 – Mutually adjusted for occupational exposures, and for education, marital status, baseline year, and long-term sickness absence spells. SHR = sub-distribution hazard, 95% CI = 95% confidence intervals.

Table S9. Associations between job exposures and early retirement, stratified by gender and occupational class at baseline

	Men				Women			
	**M1**	**M2**	**M1**	**M2**	**M1**	**M2**	**M1**	**M2**
	**Non-manual**	**Non-manual**	**Manual**	**Manual**	**Non-manual**	**Non-manual**	**Manual**	**Manual**
**Exposure**	SHR (95% CI)	SHR (95% CI)	SHR (95% CI)	SHR (95% CI)	SHR (95% CI)	SHR (95% CI)	SHR (95% CI)	SHR (95% CI)
Heavy lifting	1.015 (1.013–1.017)	1.015 (1.012–1.017)	1.004 (1.002–1.006)	1.002 (1.000–1.004)	0.993 (0.989–0.997)	0.994 (0.990–0.998)	1.000 (0.995–1.004)	0.998 (0.994–1.003)
Squatting or kneeling	0.994 (0.991–0.996)	0.993 (0.990–0.996)	1.003 (1.001–1.005)	1.001 (0.999–1.003)	1.018 (1.014–1.022)	1.017 (1.013–1.021)	1.016 (1.011–1.022)	1.014 (1.008–1.019)
Neck flexion	1.007 (1.004–1.009)	1.006 (1.004–1.009)	1.007 (1.005–1.010)	1.007 (1.005–1.010)	1.001 (0.997–1.005)	1.001 (0.997–1.005)	1.009 (1.004–1.014)	1.011 (1.006–1.016)
Forward bending	0.996 (0.994–0.998)	0.997 (0.995–0.999)	1.000 (0.998–1.003)	1.000 (0.998–1.002)	1.002 (0.998–1.005)	1.002 (0.999–1.006)	0.998 (0.994–1.002)	0.999 (0.995–1.004)
Awkward lifting	1.012 (1.010–1.015)	1.012 (1.009–1.014)	1.000 (0.998–1.003)	1.001 (0.999–1.004)	1.010 (1.007–1.013)	1.010 (1.007–1.013)	1.006 (1.002–1.010)	1.004 (0.999–1.008)
Standing or walking	1.005 (1.002–1.008)	1.005 (1.002–1.009)	1.005 (1.002–1.008)	1.004 (1.001–1.007)	0.994 (0.991–0.997)	0.995 (0.992–0.998)	1.015 (1.010–1.020)	1.016 (1.011–1.021)
Quantitative demands	0.999 (0.995–1.002)	1.000 (0.996–1.003)	1.003 (1.000–1.006)	1.004 (1.001–1.007)	1.001 (0.998–1.003)	1.000 (0.998–1.003)	0.990 (0.985–0.994)	0.990 (0.986–0.995)
Conflicting work methods	0.999 (0.996–1.001)	0.998 (0.995–1.001)	0.998 (0.996–1.001)	0.999 (0.997–1.001)	1.010 (1.006–1.014)	1.009 (1.005–1.013)	1.011 (1.006–1.015)	1.012 (1.007–1.016)
Insufficient resources	1.003 (1.001–1.006)	1.003 (1.000–1.005)	0.999 (0.997–1.002)	1.003 (1.000–1.006)	0.998 (0.993–1.002)	0.998 (0.993–1.002)	1.013 (1.008–1.018)	1.013 (1.008–1.018)
Role conflict	1.003 (0.999–1.006)	1.003 (0.999–1.006)	1.004 (1.001–1.006)	1.004 (1.001–1.006)	0.997 (0.993–1.001)	0.997 (0.993–1.001)	1.001 (0.996–1.006)	0.999 (0.994–1.004)
Low decision authority	0.995 (0.991–1.000)	0.995 (0.991–0.999)	1.005 (1.002–1.008)	1.004 (1.001–1.007)	1.007 (1.002–1.011)	1.006 (1.002–1.011)	1.007 (1.002–1.012)	1.007 (1.002–1.012)
Low skill utilization	1.000 (0.996–1.004)	1.000 (0.996–1.005)	0.992 (0.990–0.994)	0.993 (0.991–0.995)	0.992 (0.987–0.997)	0.993 (0.988–0.998)	0.992 (0.987–0.997)	0.995 (0.990–1.000)
Low skill development	0.993 (0.988–0.997)	0.993 (0.988–0.997)	1.003 (1.000–1.005)	1.002 (0.999–1.004)	1.003 (0.998–1.007)	1.003 (0.998–1.008)	0.981 (0.976–0.986)	0.983 (0.978–0.988)
Monotonous work	1.006 (1.002–1.009)	1.006 (1.003–1.009)	0.998 (0.995–1.001)	0.997 (0.994–1.000)	1.003 (0.999–1.008)	1.003 (0.999–1.008)	1.001 (0.995–1.006)	1.001 (0.995–1.006)
N	178895	178895	178895				160332	160332

M1 – Mutually adjusted for occupational exposures. M2 – Mutually adjusted for occupational exposures, and for education, marital status, baseline year, and long-term sickness absence spells. SHR = sub-distribution hazard, 95% CI = 95% confidence intervals.

Table S10. Associations between job exposures and unemployment, stratified by gender and occupational class at baseline

**Exposure**	**Men**				**Women**			
	**M1**	**M2**	**M1**	**M2**	**M1**	**M2**	**M1**	**M2**
	**Non-manual**	**Non-manual**	**Manual**	**Manual**	**Non-manual**	**Non-manual**	**Manual**	**Manual**
	SHR (95% CI)	SHR (95% CI)	SHR (95% CI)	SHR (95% CI)	SHR (95% CI)	SHR (95% CI)	SHR (95% CI)	SHR (95% CI)
Heavy lifting	0.980 (0.975–0.985)	0.982 (0.977–0.987)	0.992 (0.989–0.996)	0.997 (0.993–1.000)	1.000 (0.990–1.010)	1.005 (0.995–1.015)	0.994 (0.986–1.003)	0.997 (0.989–1.005)
Squatting or kneeling	1.001 (0.995–1.008)	1.000 (0.993–1.006)	0.996 (0.992–1.000)	0.999 (0.995–1.003)	0.979 (0.968–0.990)	0.977 (0.966–0.988)	0.979 (0.969–0.989)	0.980 (0.969–0.990)
Neck flexion	0.981 (0.975–0.987)	0.982 (0.977–0.988)	0.983 (0.979–0.987)	0.982 (0.978–0.987)	0.981 (0.971–0.991)	0.988 (0.978–0.998)	0.966 (0.955–0.978)	0.968 (0.956–0.979)
Forward bending	0.991 (0.985–0.996)	0.997 (0.991–1.003)	1.001 (0.997–1.006)	1.001 (0.997–1.006)	0.986 (0.977–0.996)	0.988 (0.978–0.997)	1.035 (1.026–1.043)	1.035 (1.026–1.043)
Awkward lifting	0.986 (0.981–0.991)	0.980 (0.975–0.985)	1.015 (1.011–1.020)	1.012 (1.008–1.017)	0.952 (0.945–0.960)	0.957 (0.949–0.965)	0.985 (0.977–0.993)	0.988 (0.980–0.996)
Standing or walking	1.013 (1.006–1.020)	1.009 (1.002–1.017)	0.987 (0.982–0.992)	0.988 (0.982–0.993)	1.016 (1.007–1.025)	1.013 (1.004–1.023)	0.970 (0.958–0.981)	0.967 (0.955–0.979)
Quantitative demands	0.988 (0.980–0.995)	0.990 (0.982–0.997)	0.992 (0.987–0.997)	0.991 (0.986–0.996)	0.997 (0.991–1.004)	0.999 (0.992–1.006)	0.998 (0.988–1.008)	1.001 (0.991–1.012)
Conflicting work methods	1.014 (1.008–1.020)	1.010 (1.004–1.016)	1.008 (1.003–1.012)	1.009 (1.005–1.014)	1.025 (1.013–1.036)	1.013 (1.002–1.025)	1.042 (1.033–1.052)	1.041 (1.031–1.051)
Insufficient resources	1.014 (1.009–1.020)	1.007 (1.001–1.013)	0.989 (0.984–0.994)	0.985 (0.979–0.990)	1.046 (1.033–1.060)	1.038 (1.025–1.052)	1.013 (1.005–1.022)	1.010 (1.001–1.019)
Role conflict	1.021 (1.013–1.030)	1.017 (1.009–1.026)	0.993 (0.988–0.998)	0.993 (0.988–0.998)	0.994 (0.982–1.006)	0.991 (0.980–1.003)	1.002 (0.992–1.011)	1.003 (0.993–1.013)
Low decision authority	0.989 (0.978–1.000)	0.987 (0.976–0.999)	0.994 (0.988–0.999)	0.997 (0.991–1.002)	0.960 (0.949–0.972)	0.964 (0.952–0.976)	0.985 (0.976–0.994)	0.988 (0.979–0.997)
Low skill utilization	1.000 (0.990–1.010)	1.002 (0.992–1.012)	1.012 (1.008–1.016)	1.011 (1.006–1.015)	0.982 (0.966–0.998)	0.984 (0.968–1.000)	0.965 (0.955–0.975)	0.967 (0.957–0.977)
Low skill development	0.997 (0.987–1.008)	0.997 (0.986–1.008)	0.991 (0.986–0.996)	0.994 (0.989–0.999)	1.031 (1.016–1.046)	1.027 (1.012–1.042)	0.998 (0.987–1.008)	0.999 (0.988–1.010)
Monotonous work	0.970 (0.963–0.977)	0.974 (0.966–0.981)	1.004 (0.998–1.010)	1.005 (0.999–1.011)	0.985 (0.973–0.997)	0.990 (0.977–1.002)	0.993 (0.982–1.005)	0.987 (0.975–0.999)
N	178895	178895			160332	160332		

M1 – Mutually adjusted for occupational exposures. M2 – Mutually adjusted for occupational exposures, and for education, marital status, baseline year, and long-term sickness absence spells. SHR = sub-distribution hazard, 95% CI = 95% confidence intervals.

Sensitivity analyses with exposures modelled categorically

Table S11. Associations between job exposures and health-related exit, stratified by gender

Exposure	M1, men SHR (95% CI)	M2, men SHR (95% CI)	M3, men SHR (95% CI)	M1, women SHR (95% CI)	M2, women SHR (95% CI)	M3, women SHR (95% CI)
Heavy lifting, ref. not exposed						
1-10 years	0.999 (0.951–1.050)	0.979 (0.931–1.030)	0.994 (0.945–1.045)	0.973 (0.941–1.006)	0.974 (0.942–1.007)	0.961 (0.929–0.994)
11-20 years	1.177 (1.114–1.243)	1.023 (0.966–1.083)	1.020 (0.964–1.080)	0.940 (0.894–0.989)	0.933 (0.886–0.982)	0.921 (0.875–0.970)
21-30 years	1.291 (1.219–1.367)	1.024 (0.964–1.087)	1.021 (0.961–1.085)	0.903 (0.834–0.978)	0.864 (0.798–0.936)	0.854 (0.787–0.925)
Squatting or kneeling, ref. not exposed						
1-10 years	1.065 (1.021–1.111)	1.035 (0.992–1.080)	1.027 (0.984–1.072)	1.005 (0.965–1.047)	1.016 (0.976–1.059)	1.014 (0.973–1.056)
11-20 years	1.131 (1.076–1.188)	1.050 (0.999–1.103)	1.037 (0.986–1.091)	0.892 (0.847–0.939)	0.924 (0.878–0.972)	0.923 (0.877–0.972)
21-30 years	1.070 (1.010–1.134)	0.960 (0.905–1.018)	0.958 (0.903–1.016)	0.859 (0.802–0.920)	0.909 (0.849–0.974)	0.905 (0.844–0.970)
Neck flexion, ref not exposed						
1-10 years	0.984 (0.941–1.029)	0.993 (0.949–1.039)	0.989 (0.945–1.035)	1.009 (0.971–1.048)	1.065 (1.025–1.108)	1.042 (1.002–1.084)
11-20 years	1.005 (0.951–1.062)	1.019 (0.964–1.078)	1.017 (0.961–1.076)	0.955 (0.909–1.003)	1.012 (0.963–1.064)	0.979 (0.931–1.029)
21-30 years	0.970 (0.907–1.037)	0.988 (0.924–1.057)	0.982 (0.918–1.050)	1.011 (0.948–1.079)	1.045 (0.979–1.116)	1.014 (0.949–1.084)
Forward bending, ref. not exposed						
1-10 years	1.060 (1.021–1.100)	1.047 (1.009–1.087)	1.028 (0.990–1.068)	1.096 (1.055–1.139)	1.034 (0.994–1.075)	1.022 (0.983–1.063)
11-20 years	1.116 (1.066–1.169)	1.118 (1.067–1.170)	1.082 (1.032–1.134)	1.276 (1.207–1.349)	1.141 (1.079–1.206)	1.101 (1.040–1.165)
21-30 years	1.163 (1.101–1.228)	1.146 (1.085–1.210)	1.118 (1.058–1.181)	1.250 (1.150–1.359)	1.129 (1.039–1.227)	1.070 (0.983–1.164)
Awkward lifting, ref. not exposed						
1-10 years	1.010 (0.966–1.056)	1.018 (0.973–1.064)	1.002 (0.958–1.048)	1.090 (1.045–1.138)	1.070 (1.025–1.117)	1.053 (1.008–1.100)
11-20 years	0.944 (0.895–0.996)	0.959 (0.909–1.011)	0.933 (0.884–0.985)	1.225 (1.159–1.295)	1.147 (1.085–1.213)	1.123 (1.061–1.188)
21-30 years	0.950 (0.891–1.013)	0.976 (0.916–1.040)	0.950 (0.891–1.012)	1.337 (1.239–1.442)	1.204 (1.116–1.298)	1.184 (1.097–1.278)
Standing or walking, ref. not exposed						
1-10 years	1.192 (1.134–1.253)	1.223 (1.163–1.286)	1.216 (1.156–1.279)	1.084 (1.031–1.140)	1.102 (1.048–1.160)	1.108 (1.053–1.167)
11-20 years	1.285 (1.215–1.360)	1.397 (1.320–1.478)	1.409 (1.331–1.491)	1.258 (1.188–1.332)	1.291 (1.218–1.368)	1.280 (1.207–1.358)
21-30 years	1.311 (1.229–1.398)	1.526 (1.430–1.628)	1.544 (1.446–1.648)	1.358 (1.267–1.456)	1.430 (1.331–1.535)	1.416 (1.317–1.522)
Quantitative demands, ref not exposed						
1-10 years	0.917 (0.887–0.947)	0.982 (0.951–1.015)	0.987 (0.955–1.021)	0.868 (0.845–0.891)	0.928 (0.903–0.954)	0.941 (0.915–0.968)
11-20 years	0.822 (0.792–0.853)	0.920 (0.886–0.954)	0.931 (0.896–0.966)	0.747 (0.718–0.778)	0.882 (0.846–0.920)	0.907 (0.869–0.946)
21-30 years	0.745 (0.713–0.779)	0.876 (0.839–0.916)	0.892 (0.853–0.933)	0.663 (0.623–0.705)	0.814 (0.763–0.868)	0.836 (0.783–0.892)
Conflicting work methods, ref. not exposed						
1-10 years	0.906 (0.873–0.940)	0.954 (0.920–0.990)	0.959 (0.923–0.996)	0.917 (0.887–0.947)	1.013 (0.980–1.047)	0.990 (0.958–1.024)
11-20 years	0.831 (0.797–0.867)	0.899 (0.862–0.937)	0.912 (0.874–0.952)	0.855 (0.811–0.901)	0.951 (0.903–1.003)	0.913 (0.865–0.962)
21-30 years	0.799 (0.760–0.840)	0.878 (0.835–0.923)	0.899 (0.854–0.946)	0.850 (0.777–0.929)	0.889 (0.814–0.970)	0.843 (0.771–0.920)
Insufficient resources, ref, not exposed						
1-10 years	0.965 (0.931–1.000)	1.005 (0.970–1.041)	1.011 (0.975–1.048)	0.978 (0.944–1.013)	0.987 (0.953–1.023)	0.993 (0.958–1.030)
11-20 years	0.907 (0.871–0.944)	0.974 (0.936–1.014)	0.973 (0.934–1.014)	0.896 (0.851–0.943)	0.959 (0.911–1.010)	0.965 (0.916–1.017)
21-30 years	0.901 (0.858–0.945)	0.967 (0.922–1.015)	0.965 (0.919–1.014)	0.801 (0.737–0.872)	0.944 (0.868–1.027)	0.952 (0.874–1.036)

Role conflict, ref. not exposed						
1-10 years	0.995 (0.956–1.035)	0.995 (0.957–1.035)	1.002 (0.963–1.043)	1.052 (1.022–1.082)	1.018 (0.989–1.047)	1.013 (0.985–1.043)
11-20 years	0.943 (0.902–0.985)	0.955 (0.915–0.998)	0.971 (0.929–1.014)	1.049 (1.002–1.098)	1.025 (0.981–1.072)	1.014 (0.969–1.061)
21-30 years	0.910 (0.864–0.957)	0.942 (0.895–0.990)	0.956 (0.909–1.007)	1.039 (0.967–1.116)	1.052 (0.980–1.129)	1.056 (0.983–1.134)
Low decision authority, ref. not exposed						
1-10 years	0.959 (0.926–0.994)	0.973 (0.939–1.008)	0.976 (0.941–1.012)	0.873 (0.837–0.911)	0.930 (0.891–0.971)	0.924 (0.885–0.965)
11-20 years	0.909 (0.871–0.948)	0.919 (0.880–0.959)	0.922 (0.883–0.963)	0.779 (0.744–0.816)	0.879 (0.838–0.921)	0.875 (0.833–0.918)
21-30 years	0.890 (0.845–0.937)	0.905 (0.859–0.953)	0.914 (0.868–0.963)	0.711 (0.674–0.750)	0.831 (0.786–0.878)	0.828 (0.782–0.875)
Low skill utilization, ref. not exposed						
1-10 years	0.990 (0.946–1.036)	1.007 (0.962–1.053)	1.008 (0.963–1.055)	1.094 (1.041–1.150)	1.017 (0.966–1.071)	1.009 (0.958–1.063)
11-20 years	0.953 (0.903–1.006)	0.966 (0.916–1.019)	0.985 (0.934–1.040)	1.139 (1.077–1.204)	1.011 (0.955–1.071)	0.989 (0.933–1.049)
21-30 years	0.921 (0.864–0.982)	0.941 (0.883–1.003)	0.955 (0.896–1.018)	1.076 (1.010–1.146)	0.959 (0.898–1.024)	0.927 (0.867–0.990)
Low skill development, ref. not exposed						
1-10 years	1.122 (1.077–1.170)	1.115 (1.070–1.163)	1.098 (1.053–1.145)	1.034 (0.990–1.079)	1.023 (0.979–1.069)	1.034 (0.989–1.081)
11-20 years	1.231 (1.171–1.295)	1.222 (1.162–1.286)	1.178 (1.120–1.240)	1.058 (1.009–1.109)	1.060 (1.011–1.113)	1.069 (1.018–1.122)
21-30 years	1.231 (1.157–1.309)	1.211 (1.138–1.288)	1.147 (1.078–1.221)	1.043 (0.987–1.104)	1.072 (1.013–1.134)	1.093 (1.032–1.158)
Monotonous work, ref. not exposed						
1-10 years	1.110 (1.062–1.160)	1.044 (0.999–1.092)	1.050 (1.004–1.098)	1.033 (0.992–1.076)	0.993 (0.953–1.035)	0.999 (0.958–1.041)
11-20 years	1.248 (1.188–1.312)	1.093 (1.039–1.149)	1.089 (1.035–1.145)	1.013 (0.964–1.065)	0.933 (0.887–0.981)	0.954 (0.906–1.004)
21-30 years	1.355 (1.281–1.432)	1.133 (1.071–1.199)	1.128 (1.066–1.194)	0.980 (0.919–1.045)	0.886 (0.830–0.945)	0.909 (0.851–0.972)
N	178895	178895	178895	160332	160332	160332

M1 – Mutually adjusted for occupational exposures. M2 – Mutually adjusted for occupational exposures, and for education, marital status and baseline year. M3 – Mutually adjusted for occupational exposures, and for education, marital status, baseline year, and long-term sickness absence spells. SHR = sub-distribution hazard, 95% CI = 95% confidence intervals.

Table S12. Associations between job exposures and early retirement, stratified by gender

Exposure	M1, men SHR (95% CI)	M2, men SHR (95% CI)	M3, men SHR (95% CI)	M1, women SHR (95% CI)	M2, women SHR (95% CI)	M3, women SHR (95% CI)
Heavy lifting, ref. not exposed						
1-10 years	1.050 (1.014–1.086)	1.041 (1.006–1.078)	1.036 (1.001–1.073)	1.066 (1.027–1.106)	1.063 (1.024–1.104)	1.069 (1.030–1.110)
11-20 years	1.103 (1.059–1.149)	1.083 (1.039–1.129)	1.081 (1.037–1.128)	1.166 (1.104–1.232)	1.167 (1.104–1.234)	1.177 (1.113–1.244)
21-30 years	1.136 (1.087–1.187)	1.123 (1.073–1.175)	1.122 (1.072–1.174)	1.183 (1.094–1.279)	1.193 (1.102–1.291)	1.205 (1.114–1.305)
Squatting or kneeling, ref. not exposed						
1-10 years	0.970 (0.936–1.004)	0.976 (0.942–1.011)	0.978 (0.944–1.013)	0.949 (0.907–0.993)	0.947 (0.904–0.991)	0.948 (0.906–0.993)
11-20 years	0.982 (0.940–1.026)	0.990 (0.947–1.034)	0.995 (0.952–1.039)	0.972 (0.917–1.029)	0.966 (0.912–1.024)	0.968 (0.913–1.026)
21-30 years	1.033 (0.980–1.087)	1.032 (0.980–1.088)	1.037 (0.984–1.092)	0.934 (0.868–1.005)	0.929 (0.863–1.000)	0.932 (0.866–1.003)
Neck flexion, ref not exposed						
1-10 years	0.989 (0.952–1.027)	0.984 (0.948–1.022)	0.987 (0.951–1.026)	1.000 (0.958–1.044)	0.986 (0.945–1.030)	0.995 (0.953–1.039)
11-20 years	1.014 (0.964–1.066)	0.996 (0.947–1.048)	0.999 (0.950–1.051)	1.057 (1.000–1.117)	1.040 (0.984–1.099)	1.054 (0.997–1.114)
21-30 years	1.046 (0.984–1.113)	1.019 (0.958–1.084)	1.026 (0.964–1.092)	1.092 (1.017–1.173)	1.085 (1.010–1.165)	1.098 (1.023–1.179)
Forward bending, ref. not exposed						
1-10 years	0.922 (0.892–0.953)	0.931 (0.901–0.962)	0.933 (0.903–0.964)	0.975 (0.934–1.018)	0.992 (0.951–1.035)	0.995 (0.953–1.038)
11-20 years	0.882 (0.844–0.921)	0.896 (0.857–0.936)	0.902 (0.864–0.943)	0.928 (0.874–0.985)	0.956 (0.900–1.015)	0.968 (0.911–1.028)
21-30 years	0.856 (0.811–0.904)	0.871 (0.825–0.919)	0.876 (0.830–0.925)	0.922 (0.848–1.002)	0.942 (0.866–1.024)	0.958 (0.882–1.042)
Awkward lifting, ref. not exposed						
1-10 years	1.010 (0.973–1.049)	1.011 (0.974–1.050)	1.016 (0.979–1.055)	0.910 (0.868–0.955)	0.909 (0.867–0.954)	0.915 (0.872–0.960)
11-20 years	1.001 (0.954–1.051)	1.001 (0.954–1.051)	1.009 (0.961–1.059)	0.791 (0.744–0.841)	0.797 (0.749–0.847)	0.807 (0.758–0.858)
21-30 years	0.961 (0.906–1.019)	0.958 (0.903–1.016)	0.965 (0.909–1.023)	0.795 (0.735–0.860)	0.808 (0.747–0.875)	0.816 (0.754–0.884)
Standing or walking, ref. not exposed						
1-10 years	1.003 (0.966–1.042)	1.000 (0.963–1.038)	1.003 (0.965–1.042)	1.029 (0.977–1.084)	1.022 (0.970–1.077)	1.022 (0.970–1.077)
11-20 years	1.004 (0.959–1.051)	1.004 (0.959–1.050)	1.006 (0.961–1.053)	1.048 (0.986–1.115)	1.041 (0.979–1.107)	1.048 (0.985–1.114)
21-30 years	1.009 (0.957–1.064)	1.008 (0.955–1.063)	1.009 (0.957–1.065)	1.076 (0.999–1.158)	1.056 (0.980–1.137)	1.066 (0.990–1.148)
Quantitative demands, ref not exposed						
1-10 years	0.990 (0.958–1.022)	0.977 (0.945–1.009)	0.975 (0.943–1.007)	0.938 (0.909–0.968)	0.929 (0.900–0.959)	0.921 (0.893–0.951)
11-20 years	1.056 (1.021–1.092)	1.035 (1.000–1.071)	1.029 (0.995–1.065)	0.996 (0.954–1.039)	0.972 (0.930–1.016)	0.962 (0.921–1.006)
21-30 years	1.152 (1.110–1.196)	1.128 (1.086–1.171)	1.120 (1.078–1.163)	1.030 (0.970–1.094)	1.004 (0.944–1.069)	0.994 (0.934–1.057)
Conflicting work methods, ref. not exposed						
1-10 years	0.985 (0.951–1.021)	0.986 (0.952–1.022)	0.983 (0.949–1.019)	1.259 (1.212–1.307)	1.207 (1.162–1.254)	1.222 (1.176–1.270)
11-20 years	0.960 (0.923–0.999)	0.957 (0.919–0.995)	0.952 (0.915–0.990)	1.396 (1.322–1.473)	1.329 (1.258–1.404)	1.356 (1.283–1.433)
21-30 years	0.955 (0.912–1.000)	0.949 (0.906–0.993)	0.942 (0.899–0.986)	1.528 (1.417–1.647)	1.466 (1.358–1.583)	1.504 (1.393–1.624)
Insufficient resources, ref, not exposed						
1-10 years	1.076 (1.040–1.114)	1.076 (1.040–1.113)	1.075 (1.039–1.113)	1.044 (1.003–1.088)	1.048 (1.006–1.092)	1.044 (1.002–1.088)
11-20 years	1.132 (1.091–1.176)	1.131 (1.089–1.174)	1.130 (1.089–1.174)	1.119 (1.059–1.181)	1.110 (1.050–1.174)	1.103 (1.043–1.166)
21-30 years	1.169 (1.120–1.222)	1.166 (1.117–1.218)	1.165 (1.116–1.217)	1.154 (1.068–1.247)	1.125 (1.040–1.218)	1.113 (1.029–1.205)
Role conflict, ref. not exposed						
1-10 years	0.945 (0.911–0.980)	0.955 (0.921–0.991)	0.953 (0.919–0.989)	0.933 (0.903–0.964)	0.953 (0.922–0.984)	0.954 (0.923–0.986)
11-20 years	0.934 (0.898–0.971)	0.953 (0.917–0.991)	0.948 (0.912–0.986)	0.973 (0.928–1.020)	0.996 (0.950–1.044)	0.998 (0.952–1.047)

21-30 years	0.927 (0.888–0.968)	0.953 (0.912–0.995)	0.948 (0.908–0.990)	0.965 (0.903–1.030)	0.975 (0.912–1.042)	0.973 (0.911–1.040)
Low decision authority, ref. not exposed						
1-10 years	1.051 (1.019–1.084)	1.047 (1.015–1.080)	1.048 (1.016–1.081)	1.018 (0.971–1.067)	1.001 (0.955–1.049)	1.000 (0.954–1.048)
11-20 years	1.161 (1.118–1.205)	1.151 (1.109–1.195)	1.152 (1.109–1.196)	1.148 (1.092–1.208)	1.108 (1.054–1.166)	1.105 (1.050–1.162)
21-30 years	1.220 (1.167–1.274)	1.198 (1.146–1.252)	1.195 (1.143–1.249)	1.249 (1.181–1.321)	1.193 (1.128–1.263)	1.189 (1.124–1.258)
Low skill utilization, ref. not exposed						
1-10 years	1.023 (0.987–1.061)	1.013 (0.977–1.050)	1.012 (0.975–1.049)	0.973 (0.927–1.021)	0.978 (0.932–1.026)	0.980 (0.934–1.029)
11-20 years	1.069 (1.023–1.118)	1.054 (1.008–1.101)	1.051 (1.005–1.098)	0.981 (0.929–1.036)	0.999 (0.946–1.055)	1.013 (0.959–1.070)
21-30 years	1.057 (1.003–1.114)	1.042 (0.989–1.099)	1.040 (0.986–1.096)	0.965 (0.909–1.025)	0.981 (0.923–1.043)	1.000 (0.940–1.062)
Low skill development, ref. not exposed						
1-10 years	0.951 (0.920–0.983)	0.965 (0.933–0.998)	0.970 (0.938–1.003)	0.975 (0.934–1.017)	0.977 (0.937–1.019)	0.972 (0.932–1.014)
11-20 years	0.953 (0.913–0.994)	0.976 (0.936–1.019)	0.988 (0.947–1.031)	0.967 (0.922–1.014)	0.967 (0.923–1.014)	0.960 (0.916–1.006)
21-30 years	1.002 (0.951–1.056)	1.035 (0.983–1.091)	1.054 (1.000–1.111)	0.969 (0.918–1.023)	0.967 (0.916–1.021)	0.956 (0.906–1.010)
Monotonous work, ref. not exposed						
1-10 years	1.022 (0.987–1.059)	1.017 (0.981–1.054)	1.015 (0.979–1.052)	1.045 (1.003–1.088)	1.048 (1.006–1.092)	1.047 (1.005–1.091)
11-20 years	0.977 (0.937–1.019)	0.973 (0.933–1.016)	0.973 (0.933–1.015)	1.160 (1.104–1.219)	1.179 (1.120–1.240)	1.169 (1.111–1.230)
21-30 years	0.959 (0.915–1.006)	0.963 (0.918–1.010)	0.964 (0.919–1.011)	1.279 (1.201–1.361)	1.305 (1.224–1.392)	1.294 (1.213–1.380)
N	178895	178895	178895	160332	160332	160332

M1 – Mutually adjusted for occupational exposures. M2 – Mutually adjusted for occupational exposures, and for education, marital status and baseline year. M3 – Mutually adjusted for occupational exposures, and for education, marital status, baseline year, and long-term sickness absence spells. SHR = sub-distribution hazard, 95% CI = 95% confidence intervals.

Table S13. Associations between job exposures and unemployment, stratified by gender

**Exposure**	**M1, men** SHR (95% CI)	**M2, men** SHR (95% CI)	**M3, men** SHR (95% CI)	**M1, women** SHR (95% CI)	**M2, women** SHR (95% CI)	**M3, women** SHR (95% CI)
Heavy lifting, ref. not exposed						
1-10 years	1.093 (1.000–1.194)	1.012 (0.926–1.107)	1.013 (0.926–1.108)	1.150 (1.071–1.236)	1.071 (0.997–1.151)	1.074 (1.000–1.154)
11-20 years	1.161 (1.053–1.280)	0.976 (0.882–1.081)	0.976 (0.882–1.080)	1.396 (1.239–1.572)	1.258 (1.118–1.416)	1.263 (1.122–1.421)
21-30 years	1.154 (1.040–1.279)	0.920 (0.825–1.026)	0.920 (0.825–1.026)	2.141 (1.751–2.617)	1.928 (1.578–2.355)	1.937 (1.586–2.367)
Squatting or kneeling, ref. not exposed						
1-10 years	1.087 (1.004–1.177)	1.048 (0.967–1.135)	1.048 (0.967–1.135)	0.934 (0.858–1.017)	0.908 (0.834–0.987)	0.908 (0.834–0.988)
11-20 years	1.119 (1.013–1.236)	1.055 (0.954–1.168)	1.055 (0.953–1.167)	0.852 (0.758–0.958)	0.824 (0.734–0.925)	0.825 (0.735–0.926)
21-30 years	1.001 (0.885–1.133)	0.929 (0.820–1.053)	0.929 (0.820–1.053)	0.812 (0.681–0.970)	0.786 (0.660–0.936)	0.786 (0.660–0.936)
Neck flexion, ref not exposed						
1-10 years	1.109 (1.020–1.206)	1.090 (1.002–1.185)	1.089 (1.002–1.184)	0.805 (0.742–0.874)	0.817 (0.753–0.887)	0.820 (0.756–0.891)
11-20 years	1.002 (0.899–1.117)	0.988 (0.886–1.103)	0.988 (0.886–1.102)	0.601 (0.533–0.677)	0.631 (0.561–0.711)	0.635 (0.564–0.715)
21-30 years	1.049 (0.917–1.199)	1.042 (0.911–1.192)	1.041 (0.910–1.191)	0.523 (0.436–0.629)	0.562 (0.468–0.674)	0.564 (0.470–0.676)
Forward bending, ref. not exposed						
1-10 years	1.035 (0.965–1.111)	1.015 (0.945–1.089)	1.014 (0.945–1.089)	0.858 (0.786–0.936)	0.843 (0.773–0.919)	0.844 (0.774–0.921)
11-20 years	1.170 (1.066–1.283)	1.138 (1.037–1.248)	1.137 (1.036–1.248)	0.668 (0.576–0.775)	0.647 (0.559–0.749)	0.649 (0.561–0.752)
21-30 years	1.372 (1.223–1.540)	1.329 (1.186–1.491)	1.329 (1.185–1.490)	0.603 (0.468–0.776)	0.581 (0.453–0.745)	0.584 (0.456–0.750)
Awkward lifting, ref. not exposed						
1-10 years	0.985 (0.909–1.068)	0.987 (0.911–1.070)	0.987 (0.911–1.070)	1.168 (1.053–1.294)	1.127 (1.016–1.250)	1.129 (1.017–1.252)
11-20 years	0.904 (0.814–1.005)	0.918 (0.826–1.020)	0.918 (0.826–1.020)	1.375 (1.197–1.581)	1.291 (1.122–1.486)	1.295 (1.125–1.490)
21-30 years	0.861 (0.756–0.980)	0.893 (0.784–1.017)	0.893 (0.784–1.016)	1.434 (1.166–1.765)	1.324 (1.076–1.630)	1.325 (1.076–1.631)
Standing or walking, ref. not exposed						
1-10 years	1.004 (0.919–1.096)	1.011 (0.925–1.105)	1.011 (0.925–1.105)	1.137 (1.012–1.278)	1.155 (1.026–1.300)	1.155 (1.026–1.300)
11-20 years	0.867 (0.784–0.960)	0.908 (0.819–1.005)	0.908 (0.819–1.005)	1.064 (0.922–1.227)	1.085 (0.939–1.255)	1.089 (0.941–1.259)
21-30 years	0.845 (0.750–0.952)	0.920 (0.816–1.038)	0.920 (0.816–1.038)	0.959 (0.796–1.155)	0.963 (0.796–1.163)	0.968 (0.801–1.170)
Quantitative demands, ref not exposed						
1-10 years	0.928 (0.873–0.987)	0.935 (0.880–0.994)	0.935 (0.880–0.994)	1.168 (1.099–1.241)	1.210 (1.139–1.287)	1.208 (1.137–1.284)
11-20 years	0.790 (0.737–0.846)	0.831 (0.776–0.890)	0.832 (0.776–0.891)	1.034 (0.937–1.141)	1.110 (1.005–1.226)	1.108 (1.003–1.224)
21-30 years	0.721 (0.666–0.781)	0.765 (0.706–0.829)	0.765 (0.706–0.830)	0.838 (0.696–1.008)	0.921 (0.764–1.111)	0.921 (0.764–1.110)
Conflicting work methods, ref. not exposed						
1-10 years	1.043 (0.971–1.121)	1.041 (0.968–1.119)	1.041 (0.968–1.119)	0.726 (0.677–0.778)	0.713 (0.665–0.765)	0.716 (0.667–0.768)
11-20 years	1.034 (0.953–1.121)	1.053 (0.971–1.143)	1.054 (0.971–1.143)	0.620 (0.548–0.703)	0.604 (0.534–0.683)	0.607 (0.537–0.687)
21-30 years	0.917 (0.829–1.013)	0.940 (0.850–1.040)	0.941 (0.850–1.040)	0.437 (0.334–0.571)	0.403 (0.311–0.523)	0.406 (0.313–0.527)
Insufficient resources, ref, not exposed						
1-10 years	0.975 (0.913–1.040)	0.980 (0.918–1.047)	0.980 (0.918–1.047)	0.885 (0.819–0.956)	0.931 (0.862–1.006)	0.930 (0.861–1.005)
11-20 years	0.840 (0.780–0.905)	0.857 (0.796–0.924)	0.857 (0.796–0.924)	0.700 (0.618–0.793)	0.775 (0.684–0.878)	0.772 (0.682–0.875)
21-30 years	0.599 (0.544–0.660)	0.615 (0.558–0.678)	0.615 (0.558–0.678)	0.713 (0.575–0.884)	0.809 (0.653–1.002)	0.806 (0.650–0.999)
Role conflict, ref. not exposed						
1-10 years	1.104 (1.023–1.190)	1.115 (1.033–1.202)	1.115 (1.034–1.203)	1.117 (1.047–1.191)	1.090 (1.022–1.161)	1.090 (1.023–1.162)
11-20 years	1.052 (0.966–1.145)	1.081 (0.993–1.176)	1.081 (0.994–1.177)	1.155 (1.025–1.301)	1.136 (1.011–1.277)	1.138 (1.012–1.279)

21-30 years	1.012 (0.914–1.121)	1.064 (0.961–1.178)	1.064 (0.962–1.178)	1.064 (0.829–1.365)	1.108 (0.868–1.414)	1.107 (0.867–1.414)
Low decision authority, ref. not exposed						
1-10 years	1.080 (1.013–1.151)	1.012 (0.949–1.080)	1.012 (0.949–1.080)	0.848 (0.760–0.946)	0.869 (0.778–0.971)	0.869 (0.778–0.972)
11-20 years	1.171 (1.086–1.262)	1.053 (0.977–1.136)	1.053 (0.977–1.136)	0.652 (0.578–0.735)	0.696 (0.616–0.788)	0.697 (0.616–0.788)
21-30 years	1.233 (1.128–1.348)	1.061 (0.970–1.160)	1.061 (0.970–1.160)	0.443 (0.385–0.511)	0.484 (0.418–0.560)	0.484 (0.418–0.561)
Low skill utilization, ref. not exposed						
1-10 years	1.159 (1.067–1.259)	1.098 (1.010–1.194)	1.098 (1.010–1.194)	1.484 (1.265–1.740)	1.336 (1.135–1.574)	1.340 (1.138–1.578)
11-20 years	1.091 (0.992–1.200)	1.020 (0.926–1.124)	1.021 (0.926–1.125)	1.310 (1.103–1.554)	1.157 (0.970–1.381)	1.163 (0.975–1.387)
21-30 years	1.155 (1.032–1.291)	1.077 (0.962–1.207)	1.078 (0.963–1.207)	1.302 (1.075–1.577)	1.125 (0.923–1.370)	1.131 (0.929–1.378)
Low skill development, ref. not exposed						
1-10 years	0.989 (0.921–1.062)	0.987 (0.917–1.062)	0.986 (0.917–1.061)	1.051 (0.936–1.181)	1.041 (0.923–1.174)	1.040 (0.922–1.173)
11-20 years	0.885 (0.810–0.967)	0.895 (0.817–0.980)	0.894 (0.817–0.979)	1.023 (0.900–1.163)	1.028 (0.900–1.174)	1.027 (0.900–1.173)
21-30 years	0.704 (0.629–0.788)	0.710 (0.633–0.796)	0.709 (0.632–0.795)	1.069 (0.919–1.243)	1.090 (0.932–1.274)	1.089 (0.932–1.273)
Monotonous work, ref. not exposed						
1-10 years	1.207 (1.112–1.309)	1.170 (1.078–1.270)	1.170 (1.078–1.270)	1.513 (1.316–1.740)	1.323 (1.151–1.520)	1.322 (1.150–1.519)
11-20 years	1.281 (1.171–1.401)	1.205 (1.101–1.318)	1.205 (1.101–1.318)	2.046 (1.754–2.388)	1.716 (1.470–2.002)	1.709 (1.464–1.994)
21-30 years	1.425 (1.291–1.573)	1.364 (1.236–1.507)	1.364 (1.235–1.506)	2.499 (2.071–3.015)	2.013 (1.666–2.431)	2.001 (1.657–2.417)
N	178895	178895	178895	160332	160332	160332

M1 – Mutually adjusted for occupational exposures. M2 – Mutually adjusted for occupational exposures, and for education, marital status and baseline year. M3 – Mutually adjusted for occupational exposures, and for education, marital status, baseline year, and long-term sickness absence spells. SHR = sub-distribution hazard, 95% CI = 95% confidence intervals.

References

Hanvold, T. N., et al. (2019). "Mechanical and psychosocial work exposures: the construction and evaluation of a gender-specific job exposure matrix (JEM)." Scandinavian journal of work, environment & health (3): 239-247. https://doi.org/10.5271/sjweh.3774
